# Synthesis, Biological Evaluation and Molecular Docking Studies of 5-Indolylmethylen-4-oxo-2-thioxothiazolidine Derivatives

**DOI:** 10.3390/molecules27031068

**Published:** 2022-02-05

**Authors:** Volodymyr Horishny, Athina Geronikaki, Victor Kartsev, Vasyl Matiychuk, Anthi Petrou, Pavel Pogodin, Vladimir Poroikov, Theodora A. Papadopoulou, Ioannis S. Vizirianakis, Marina Kostic, Marija Ivanov, Marina Sokovic

**Affiliations:** 1 Department of Chemistry, Danylo Halytsky Lviv National Medical University, Pekarska 69, 79010 Lviv, Ukraine; vgor58@ukr.net; 2 Department of Pharmaceutical Chemistry, School of Pharmacy, Aristotle University of Thessaloniki, 54124 Thessaloniki, Greece; anthi.petrou.thessaloniki1@gmail.com; 3 InterBioScreen, Chernogolovka, 142432 Moscow Region, Russia; vkartsev@ibscreen.chg.ru; 4 Department of Chemistry, Ivan Franko National University of Lviv, Kyryla i Mefodia 6, 79005 Lviv, Ukraine; v_matiychuk@ukr.net; 5 Institute of Biomedical Chemistry, Pogodinskaya Street 10 Bldg.8, 119121 Moscow, Russia; pogodinpv@gmail.com (P.P.); vladimir.poroikov@ibmc.msk.ru (V.P.); 6 Department of Pharmacology, School of Pharmacy, Aristotle University of Thessaloniki, 54124 Thessaloniki, Greece; theodpapad@pharm.auth.gr (T.A.P.); ivizir@pharm.auth.gr (I.S.V.); 7 Department of Life and Health Sciences, University of Nicosia, Nicosia CY-1700, Cyprus; 8 Mycological Laboratory, Department of Plant Physiology, Institute for Biological Research, Siniša, Stankovic-National Institute of Republic of Serbia, University of Belgrade, Bulevar Despota Stefana 142, 11000 Belgrade, Serbia; marina.kostic@ibiss.bg.ac.rs (M.K.); marija.smiljkovic@ibiss.bg.ac.rs (M.I.); mris@ibiss.bg.ac.rs (M.S.)

**Keywords:** 4-oxo-2-thioxothiazolidine, antibacterial, antifungal, microdilution method, biological activity prediction, docking, MurB, CYP51

## Abstract

Background: Infectious diseases represent a significant global strain on public health security and impact on socio-economic stability all over the world. The increasing resistance to the current antimicrobial treatment has resulted in the crucial need for the discovery and development of novel entities for the infectious treatment with different modes of action that could target both sensitive and resistant strains. Methods: Compounds were synthesized using the classical organic chemistry methods. Prediction of biological activity spectra was carried out using PASS and PASS-based web applications. Pharmacophore modeling in LigandScout software was used for quantitative modeling of the antibacterial activity. Antimicrobial activity was evaluated using the microdilution method. AutoDock 4.2^®^ software was used to elucidate probable bacterial and fungal molecular targets of the studied compounds. Results: All compounds exhibited better antibacterial potency than ampicillin against all bacteria tested. Three compounds were tested against resistant strains MRSA, *P. aeruginosa* and *E. coli* and were found to be more potent than MRSA than reference drugs. All compounds demonstrated a higher degree of antifungal activity than the reference drugs bifonazole (6–17-fold) and ketoconazole (13–52-fold). Three of the most active compounds could be considered for further development of the new, more potent antimicrobial agents. Conclusion: Compounds **5b** (Z)-3-(3-hydroxyphenyl)-5-((1-methyl-1*H*-indol-3-yl)methylene)-2-thioxothiazolidin-4-one and **5g** (Z)-3-[5-(1*H*-Indol-3-ylmethylene)-4-oxo-2-thioxo-thiazolidin-3-yl]-benzoic acid as well as **5h** (Z)-3-(5-((5-methoxy-1*H*-indol-3-yl)methylene)-4-oxo-2-thioxothiazolidin-3-yl)benzoic acid can be considered as lead compounds for further development of more potent and safe antibacterial and antifungal agents.

## 1. Introduction

During the last century, several dozen infections have grown and affected the health of millions of people all over the world [1]. In addition to emerging infections, antimicrobial resistance accounts for at least 50,000 deaths each year in Europe and the United States and it is expected that drug resistant infections will be responsible for even larger losses worldwide in the near future [2,3]. Resistant pathogens threaten patients in medical facilities and may emerge in the general population due to the irrational use of antimicrobial agents [4]. Moreover, microbes can transit to the biofilm-growing form to mitigate the harsh environmental conditions or tolerate the presence of a drug. Existing antimicrobial treatment often fails to prevent or eliminate such biofilms [5,6]. 

Unfortunately, only few novel classes of antibacterial agents (i.e., oxazolidinones, pleuromutilins, tiacumicins, diarylquinolines lipopeptides and streptogramins) have been marketed in the recent decades to solve these problems. Most of them are for the management of Gram-positive bacterial infections [7,8]. However, drug discovery and development is still one of the major ways to ease the burden of microbial infections. Thus, novel molecules with antimicrobial activity are needed and knowledge on their activity profile including both bacterial and molecular targets and off-targets is needed too to provide the possibility to fix such problems as emerging novel infections, drug resistance and tolerance in a rational way.

**Figure 1:**
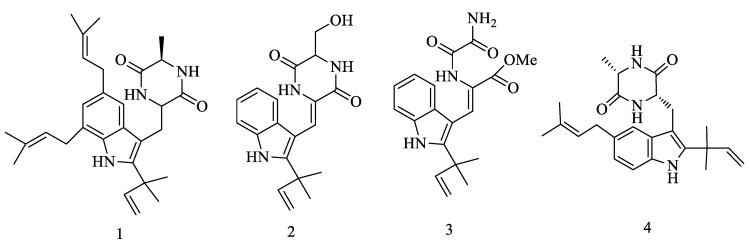
Structure of some indole alkaloids.

Among the natural compounds, there are some indole alkaloids, such as echinulin (1), cristatumin A (2), cristatumin D (3) and tardioxopiperazine A (4) from *Eurotium cristatum* EN-220 that were able to inhibit the growth of *Escherichia coli* and *S. aureus* bacteria [9] (Figure 1).

**Figure 2:**
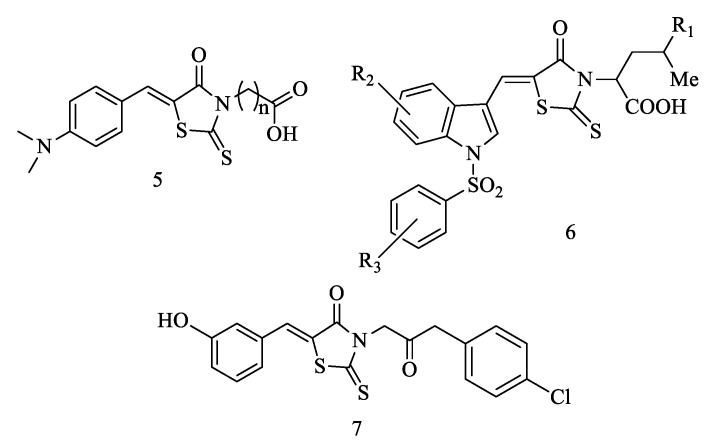
Structure of some rhodanine analogues.

It is known that rhodanin is one of the most notorious key materials for the development of effective antibiotics. The favorable antimicrobial activity of rhodanines is due to the similarity of their structure with the chemical structure of penicillin, which has been proved by several researchers [10,11,12,13]. Rhodanine-3-alkanecarboxylic acid derivatives with p-*N,N*-benzylidenedialkyl (phenyl)amine moieties on benzene ring **5** were found to be active against staphylococcus, micrococcus and streptococcus strains [14]. Rhodanines bearing *N*-arylsulfonylindole fragment derivatives **6** exhibited inhibitory activity against *S. aureus* including methicillin resistant strains (MRSA) [15], while rhodanine **7** was found to be potent against methicillin-resistant *Staphylococcus aureus*, *Staphylococcus epidermidis*, *Staphylococcus aureus*, *Enterococcus* sp. and Mycobacteria (Figure 2). 

**Figure 3:**
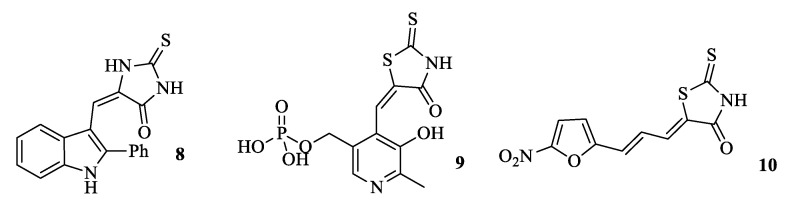
Structure of some synthetic rhodanic analogues.

It is also known that synthetic thiohydantoin (rhodanine) analogues **8**, **9**, in addition to antitumor and anti-HIV activity, exhibit pronounced antibacterial properties [16], along with some hetarylidene thiazolidines containing pyridine 9 [17] and furan 10 [12,18] fragments in the side chain being beta-lactamase inhibitors (Figure 3).

Furthermore, 5-arylidene derivatives of rhodanines were found to possess various types of activity, in particular antitumor [19], antiviral [20,21], anti-inflammatory, antidiabetic [22,23,24], antioxidant [25], LOX and cholinesterase inhibitory activities [26,27], as well as aldose reductase inhibitor activity [28]. There are many references in the literature regarding antimicrobial activity of rhodanine derivatives [14,29,30,31,32].

Prompted by everything mentioned above, as well as based on our previous results [33,34], we designed and synthesized new derivatives incorporating two pharmacophores in the frame of one molecule, indole and thiazolidinone, using a hybridization approach. The aim of this approach is mainly to improve the activity profile and reduce undesired side effects.

As is known, rhodanine derivatives are synthesized by several methods, in particular by dithiocarbamate, bis(carboxymethyl)trithiocarbonate (the Holmberg method), and thiocyanate [35]. The starting 3-arylrhodanines were synthesized using the Holmberg method, making it possible to obtain compounds based on arylamines in high yields and sufficient purity, avoiding the formation of thiourea impurities. To obtain the target products, the interaction of 3-arylrhodanines with aldehydes under the conditions of the Knoevenagel reaction was used [36].

## 2. Results and Discussion

### 2.1. Chemistry

**Scheme 1:**
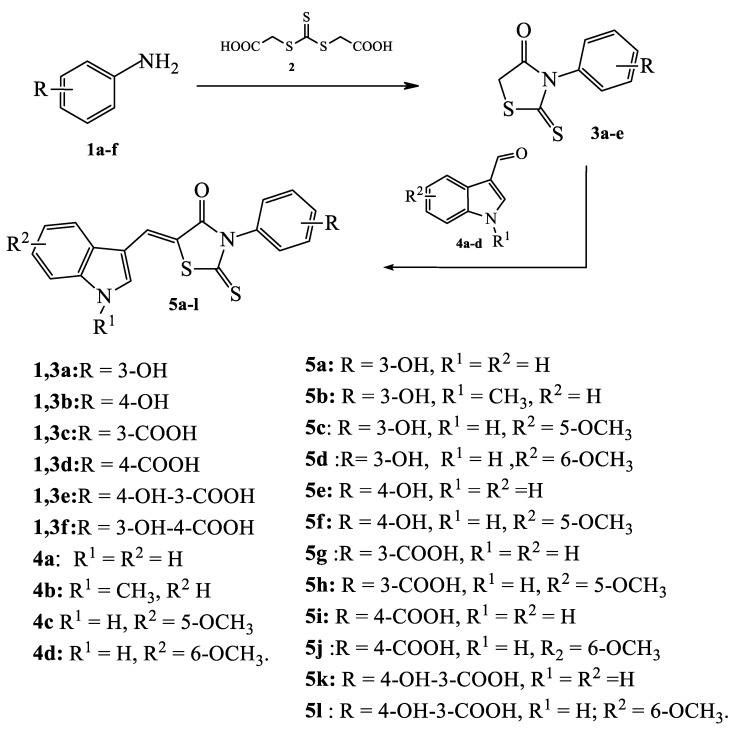
Synthesis of titled compounds.

The starting materials for the synthesis of the described products were 3-aryl-2-thioxothiazolidin-4-ones **3a**–**e**. They were obtained by the reaction of aromatic amines **1a**–**e** with bis (carboxymethyl) trithiocarbonate **2** (Scheme 1). In the second step, the 3-aryl-2-thioxothiazolidin-4-ones further undergo Knovenagel condensation with 1*H*-indole-carbaldehyde to give the title compounds **5a**–**l**. We used the optimized procedure to do this; the details of the procedure are given in Table 1. Unfortunately, we were not able to obtain 2-hydroxy-4-(4-oxo-2-thioxo-thiazolidin-3-yl)-benzoic acid **3f** using 4-amino-2-hydroxybenzoic acid as the starting material **1f**, since spontaneous decarboxylation with the formation of **3a** occurred under the reaction conditions.

We studied the interaction of 3-aryl-2-thioxothiazolidin-4-ones **3a**–**e** with 1*H*-indole-3-carbaldehydes **4a**–**d**. It was found that, when these reagents are boiled in acetic acid in the presence of ammonium acetate, 3-aryl-5-(1*H*-indol-3-ylmethylene) -2-thioxothiazolidin-4-ones **5a**–**l** are formed in good yields (Scheme 1).

An analysis of the results of obtaining the initial three and target five substances makes it possible to detect a correlation between the substituents’ nature and the products’ yields. Thus, para-substituents in the aromatic ring in position 3 of thiazolidine have a positive effect on the yields compared to the corresponding meta-substituents. In this case, the nature of the substituent also matters. Hydroxyl substituents have a more favorable effect on yields than carboxyl substituents.

The structures of all synthesized compounds were confirmed by ^1^H and ^13^C NMR spectroscopy. In the ^1^H NMR spectra, signals of all protons were present in regions that correspond to the structure of the obtained compounds. In particular, the signals of the methylene group of 3-aryl-2-thioxothiazolidin-4-ones **3a**–**e** are in the range 4.33–4.40 ppm. Signals of the methylidene proton of 3-aryl-5-(1*H*-indol-3-ylmethylene)-2-thioxothiazolidin-4-ones **5a**–**l** resonate at 7.99–8.17 ppm, which indicates the Z configuration of the double bond at the 5th position of the thiazolidine ring [37,38]. The signals of hydroxy groups were observed at 9.49–9.87 ppm, while those of the NH group were observed at 11.97–12.37 ppm. The indol ring protons appeared in the aromatic area in the range of 6.73–8.15 ppm. The detailed explanation is given in the experimental part.

### 2.2. PASS-Based In Silico Assessment of Compounds’ Activity

Activities of twelve chemical structures had been assessed using PASS [39] and PASS-based web applications to predict antifungal, antibacterial, and kinase inhibitory activity [37,38]. 

PASS predicted some activities associated with antibacterial and antifungal effects. The antibacterial activity itself has been predicted for each of the 12 structures. Pa-Pi values were in the range from 0.003 to 0.577. According to the PASS assessments, the most probable mechanism of antibacterial effect of studied chemical compounds are:

(1) Inhibition of Enoyl-[acyl-carrier-protein] reductase (predicted for 12 compounds with Pa-Pi values in range from 0.136 to 0.577).
(2) Inhibition of (R)-Pantolactone dehydrogenase (predicted for five compounds with Pa-Pi values in the range from 0.02 to 0.247).
(3) Either with lower Pa-Pi values or for the smaller number of compounds, the several other mechanisms were predicted: inhibition of D-Ala-D-Ala ligase and histidine kinase, antagonism with para-aminobenzoic acid, and antagonism with human tumor necrosis factor-alpha.

AntiBac-Pred assessed the probable activity for 12 structures against multiple bacterial strains and species with Pa-Pi values ranging from 0.0001 to 0.387. Overall, some bacteria were predicted as targets for each of the 12 molecules. However, Pa-Pi values were low, only for seven compounds out of 12 did they exceeded 0.3. These assessments were obtained for the activity of compounds against the Bacillus subtilis subsp. subtilis str. 168.

PASS predicted general antifungal activity as well as some putative mechanisms of antifungal action (Heat shock protein 90 antagonist, Kinase inhibitor) for all 12 compounds with Pa-Pi = 0.005 ÷ 0.612. Application of AntiFun-Pred, however, did not allow identification of the specificity of antifungal action against the particular fungi strains.

Among the PASS-predicted mechanisms of antibacterial and antifungal activities, inhibition of some kinases was identified. To estimate the action on the studied compounds on 20 kinases in more detail, we applied the KinScreen web application. As a result, for all twelve compounds, inhibitory activity was predicted against one or more kinases with Pa-Pi > 0.5. For six compounds, inhibition of three kinases (serine/threonine-protein kinase haspin, serine/threonine-protein kinase Nek11, and serine/threonine-protein kinase SRPK1) was estimated with Pa-Pi ≥ 0.7. Therefore, the studied compounds’ antibacterial action may be also due to kinases’ inhibition, including those belonging to the host (human) organism.

Taken together, the results of PASS-based activity evaluation suggest that the studied compounds may exhibit antibacterial and antifungal activities. Relatively low, but not negative, Pa-Pi, values obtained with PASS Online, AntiBac-Pred and AntiFun-Pred indicate that either the studied compounds (1) have a significant structural novelty compared to the compounds from the available training sets or (2) structurally similar compounds may be found among both active and inactive examples in the training set. Experimental studies in antibacterial assays could clarify the selectivity of the compounds’ action on particular bacteria. In turn, docking of the studied chemical structures to the targets could clarify molecular mechanism of their action.

### 2.3. Pharmacophore Modelling Study

Taking into account the well obtained antibacterial results against *S. aureus* of the synthesized compounds of our previews work [34], we selected these molecules for the pharmacophore modelling study in order to search for more active compounds. The structures of the training set (**1**–**9**) and test set compounds (**10**–**14**) are displayed in Table 2. In order to evaluate the common features of these compounds, crucial for the antibacterial activity, the LigandScout program was used.

**Figure 4:**
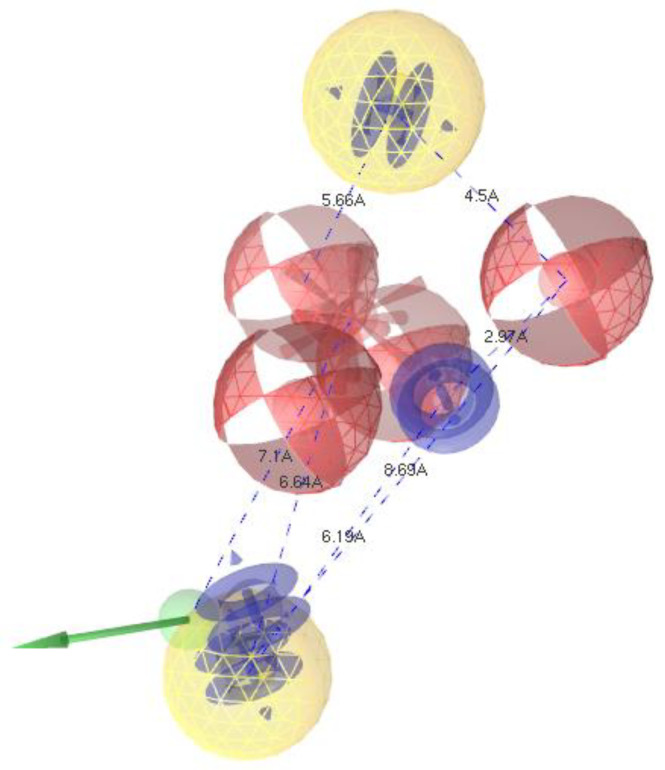
The proposed pharmacophore model of antibacterial activity (in yellow color the hydrophobic (H), red hydrogen bond acceptor (HBA), green hydrogen bond donor (HBD), red star positive ionizable (PI) and blue aromatic (AR) features).

Conformation generation within 20 kcal/mol energy range were generated and submitted to the alignment procedure. Pharmacophore run resulted in the generation of 10 hypothesis models categorized by their rank score and mapping into all training set molecules (Table 3). From all models, model-1 was selected as the best hypothesis for further analysis based on its highest rank score and mapping (Figure 4). Model-1 contains twelve features, two hydrophobes (H), four hydrogen bond acceptors (HBA), four aromatics (AR) one positive ionizable features (PI) and one hydrogen bond donor (HBD).

**Figure 5:**
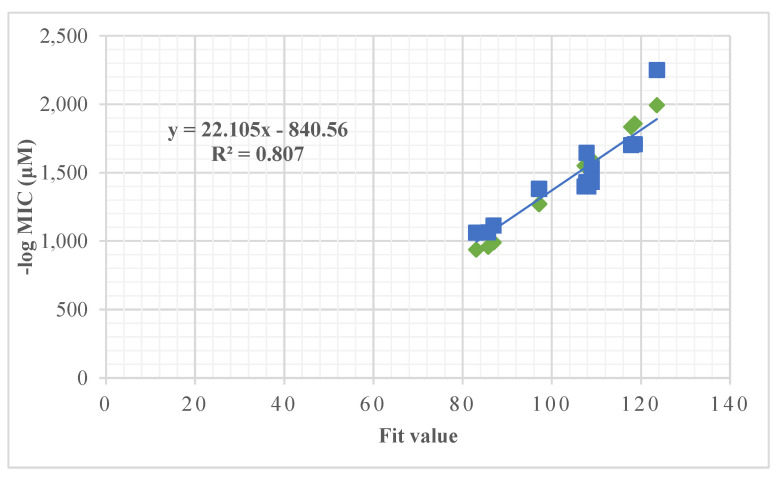
Negative logarithm of the MIC in μM (dependent value) against the LigandScout program output fit value (independent value).

All compounds (training and test sets) were mapped onto model-1 with scoring of the orientation of a mapped compound within the hypothesis features using a “fit value” score. As a primary validation of model-1, mapping of all compounds revealed a good correlation between the biological activity and the fit value score (Table 4). The compounds with the higher antibacterial activity showed a range of fit values of 123.59–108.35, while the rest showed a range of fit values of 97.23–85.10. This primary correlation encouraged us to generate a linear model based on “fit value” in order to predict the antibacterial activity of the understudy compounds. This model (Equation (1), Figure 5) showed good statistics with R^2^ = 0.807. Thus, based on these findings, we use this linear model in order to calculate the activity of the tested compounds (**5a**–**5l**) (Table 5).

−logMIC(μM) = 22.105 fitvalue–840.56, R = 0.898, R^2^ = 0.807
*n* = 14, st error: slope = 3.120, Y-intercept = 327.5, 1/slope = 0.04524 (1)

where *n* is the number of compounds and R is the multiple correlation coefficient.

**Figure 6:**
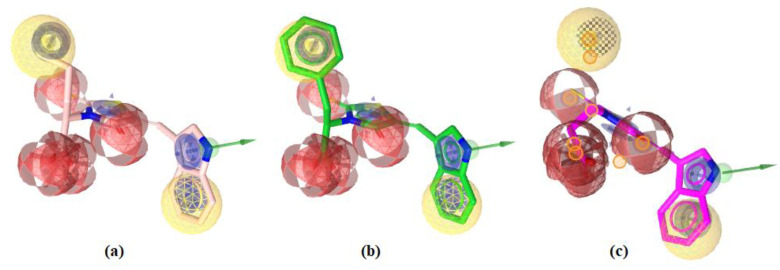
(**a**) Best aligned pose of compound **1** (MIC 0.56 μM × 10^−2^) superposed with model-1. (**b**) Best aligned pose of compound **2** (MIC 1.96 μM × 10^−2^). (**c**) Best aligned pose of compound **14** (MIC 8.66 μM × 10^−2^).

The best aligned poses of the most active compounds **1** and **2** and the less active **14**, superposed with model-1, are presented in Figure 6. As is obvious, some structural features play a key role for the activity. The 1*H*-indole moiety is thought to be critical for activity. Additionally, the absence of hydrophobic groups can partially explain their lack of activity. The other features that are common for all compounds are three HBA features and the positive ionizable interaction of carboxyl group.

As we mention above, we use this model in order to predict the antibacterial activity of synthesized compounds **5a**–**5l**. Results are presented in Table 4 and revealed that compounds **5a**–**c** have the highest fit value score and therefore probably the highest activity. Indeed, the experimental data revealed that the predicted by linear model and the experimental MIC of each compound were in the same range (Table 5). 

### 2.4. Biological Evaluation

#### 2.4.1. Antibacterial Action

Compounds **5a**–**5l** were tested for antimicrobial activity against the panel of Gram-positive, Gram-negative bacteria as well as eight fungal species.

The determination of minimal inhibitory and minimal bactericidal/fungicidal activity was performed using the microdilution method. The evaluation revealed that all compounds showed antibacterial activity with MIC at 3.78–12.77 µmol/mL × 10^−2^ and MBC at 4.09–17.03 µmol/mL × 10^−2^ (Table 6).

The antibacterial potency of tested compounds can be presented as follows: **5b** > **5h** > **5g** > **5c** > **5l** > **5a** > **5j** > **5k** > **5i** > **5d** > **5f** > **5e**. The best activity was observed for compound **5b** with MIC and MBC at 3.00–12.28 µmol/mL × 10^−2^ and 4.09–16.31 µmol/mL × 10^−2^, respectively, while compound **5e** displayed the lowest activity with MIC in the range of 3.12–12.77 µmol/mL × 10^−2^ and MBC at 4.26–17.03 µmol/mL × 10^−2^. It should be noticed that bacteria, in general, showed different sensitivity towards the compound tested.

Thus, the sensitivity of *S. aureus*, the most sensitive bacterium among Gram-positive bacteria, can be presented as: **5b** > **5h** > **5a** > **5c** > **5g** > **5l** > **5j** > **5d** > **5k** > **5f** > **5e** > **5i**. Some similarities in sensitivity with *S. aureus* was observed for *En. cloacae*, the most sensitive Gram-negative bacterium: **5b** > **5e** > **5h** > **5a** > **5c** > **5g** > **5l** > **5f** > **5d** > **5j** > **5k** > **5i**. Sensitivity towards compounds of *B. cereus*, the most resistant Gram-positive bacterium appeared to be completely different: **5g** > **5d** = **5l** > **5h** = **5j** > **5a** = **5k** > **5f** > **5c** > **5i** > **5b** > **5e**, while for the most resistant Gram-negative bacterium, *E. coli* the sensitivity can be presented as: **5k** > **5a** > **5d** = **5l** > **5h** = **5j** > **5e** > **5c** = **5f** > **5g** = **5i** > **5b**. In this case too some similarities were observed. Thus, both bacteria *B. cereus* and *E. coli* exhibited the same sensitivity to compounds **5h** and **5j**, as well as **5d** and **5l**.

Compounds **5a**–**5d** and **5g** exhibited good activity against *En. cloacae* with MIC values in the range of 3.12–3.94 µmol/mL × 10^−2^ and MBC at 4.09–7.89 µmol/mL × 10^−2^. Some of these compounds (**5a**, **5b**, **5g**) also showed good activity against *P. aeruginosa* with MIC and MBC at 3.0–3.94 µmol/mL × 10^−2^ and 4.26–7.89 µmol/mL × 10^−2^, respectively, while compound **5b** and **5g** displayed good activity against *S. aureus* as well (MIC/MBC at 3–3.94/4.09–7.89 µmol/mL × 10^−2^).

*S. aureus* was found to be the most sensitive bacteria among all bacteria involved in the study, whereas *E. coli* was the most resistant one. 

Precisely, for Gram-positive bacteria, the range of MIC and MBC was 3.00–12.77 µmol/mL × 10^−2^ and 7.57–17.03 µmol/mL × 10^−2^, respectively, while for Gram negative bacteria the MIC and MBC were in range from 3.78 to 14.07 µmol/mL × 10^−2^ and 7.57 to 28.14 µmol/mL × 10^−2^, respectively, indicating that tested compounds are more potent against Gram-positive bacteria than against Gram-negative bacteria.

All compounds were more potent than ampicillin (MIC at 24.8–74.4 µmol/mL × 10^−2^ and MBC at 37.2–124.0 µmol/mL × 10^−2^) and almost all compounds were more potent than streptomycin. This is true for almost all bacterial species, except for *B. cereus* and *S. typhimurium*.

Four the most active compounds were tested against three resistant strains: methicillin-resistant *S. aureus*, *MRSA*, *P. aeruginosa* and *E. coli* (Table 7). Two of these strains MRSA and *P. aeruginosa* belong to ESKAPE pathogens (*Enterococcus faecium*, *Staphylococcus aureus*, *Klebsiella pneumoniae*, *Acinetobacter baumannii*, *Pseudomonas aeruginosa*, and *Enterobacter* species). Tackling the problem of MRSA is a top priority for public health systems worldwide. As far as *P. aeruginosa* is concerned, the incidences of diseases caused by it are fairly low in the general population, but are higher in hospital inpatients, especially those which are immunocompromised.

All compounds appeared to be more potent against MRSA than ampicillin, displaying better bactericidal activity also against resistant strains *P. aeruginosa* and *E. coli* than ampicillin, which did not show any bactericidal effect. These compounds were tested also for their effect on biofilm formation. The antibiofilm effect of selected compounds was less promising compared to their potential to inhibit growth of planktonic bacterial cells. Compounds **5b** and **5g** had a slight effect on biofilm inhibition, 11.59 and 18.19% inhibition, respectively (Table 7).

The structure–activity relationship revealed that the presence of the methyl group in the indole ring as well as the hydroxy group in position 3 of benzene ring (**5b**) of (Z)-3-(3-hydroxyphenyl)-5-((1-methyl-1*H*-indol-3-yl)methylene)-2-thioxothiazolidin-4-one is beneficial for antibacterial activity. Displacement of the methyl group by hydrogen in the indole ring and at the same time introduction of the 5-MeO group to the indole ring from one hand and replacement of the 3-OH group in the benzene ring by 3-COOH gave compound **5h**, second in the order of activity. On the other hand, removal of methyl as a substituent on the nitrogen and the 5-OMe group of the indole ring resulted in compound **5g**, with decreased activity being the third one in the order of activity of compounds. In general, among 5-OMe- indole derivatives more favorable for activity is the presence of 3-COOH (**5h**) than the 3-OH group (**5c**) in benzene ring. On the other hand, shifting of the 3-OH group to position 4 of benzene ring was negative, leading to one of the less potent compounds **5f**, whereas removal of the 5-MeO group of compounds **5f** appeared to be detrimental, resulting in the less active compound **5e**. In case of 6-OMe derivatives, the presence of 4-OH, 3-COOH substitution is (**5l**) beneficial, while the opposite 3-OH, 4-COOH (**5d**) was negative. On the other hand, substitution in position 4 of the benzene ring with the hydroxy group resulted in compound (**5j**), with increased activity compared to **5d**. From all these compounds mentioned above, it is clear that the antibacterial activity of designed and synthesized compounds depends not only on substituents and their position in the indole ring but also on substituents and their position in the benzene ring.

All the compounds, except **5g**, were additive with streptomycin (FICI 1.5, Table 8), implying that efficient combination with this antibiotic might be further developed. Compound **5g** was indifferent in combination with streptomycin (FICI 2).

#### 2.4.2. Efficient *P. aeruginosa* Bactericidal Effect after 1 h

**Figure 7:**
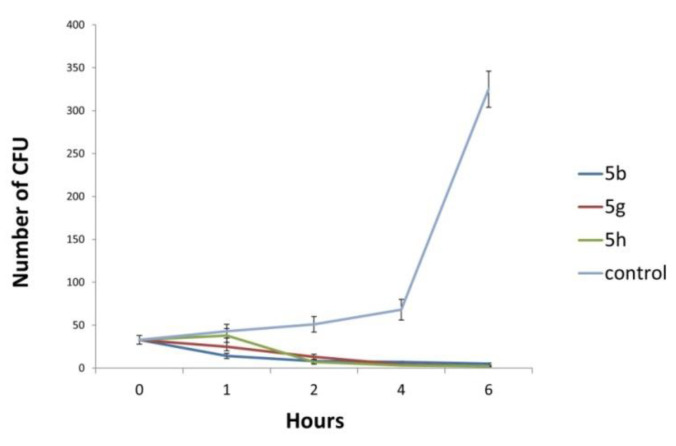
Number of *P. aeruginosa* CFUs after different time intervals of antimicrobial treatment with the MBC of tested compounds.

Application of the selected compounds has significantly reduced the number of viable *P. aeruginosa* colonies (Figure 7). After 2 h of application, the number of viable *P. aeruginosa* colonies was reduced for more than 84% (**5b** and **5h**) and more than 74% (**5g**). All of the examined compounds, after 6h of treatment, reduced the number of *P. aeruginosa* CFU by more than 98%.

It should be mentioned that even the less active compound **5b** appeared to be able to reduce the number of *P. aeruginosa* CFUs.

#### 2.4.3. Antifungal Action

The ability of compounds to inhibit fungi growth is presented in Table 9 and follows the order **5k** > **5l** > **5f** > **5g** > **5a** > **5h** > **5i** > **5c** > **5j** > **5b** > **5d** > **5e**. The minimum inhibitory concentration of compounds was in the range of 0.97–34.05 mmol/mL × 10^−2^, while the minimal fungicidal concentration (MFC) varied from 1.95 to 68.1 µmol/mL × 10^−2^. The best antifungal activity was achieved by compound **5k** with MIC and MFC in range of 1.88–3.52 µmol/mL × 10^−2^ and 3.52–7.03 µmol/mL × 10^−2^, respectively. The lowest activity was observed for compound **5e** with an MIC at 3.12–34.05µmol/mL × 10^−2^ and MFC at 4.26–68.10µmol/mL × 10^−2^.

Ketoconazole demonstrated antifungal potency with an MIC in the range of 38–475 µmol/mL × 10^−2^ and MFC at 95–570 µmol/mL × 10^−2^, whereas MIC and MFC of bifonazole were at 48–64 mmol/mL × 10^−2^ and 64–80 µmol/mL × 10^−2^, respectively.

The sensitivity of fungi towards tested compounds was different. The sensitivity of the most vulnerable fungi *T. viride* to the compounds can be presented as follows: **5h** > **5d** > **5a-** = **5k** > **5f** > **5g** = **5i** > **5b** > **5e** > **5c** > **5l** > **5j**, while the sensitivity of the most resistant one, *A. fumigatus*, differs significantly: **5l** > **5a** = **5k** > **5g** = **5i** > **5b** > **5j** > **5h** > **5c** = **5f** > **5e**. Species belonging to the Aspergillus genus are among the most frequent causes of human fungal infections, associated with significant mortality. A member of this genus, *A. fumigatus*, is estimated as a cause of 90% of aspergillus infections [40], especially in individuals with immunodeficiency. Compound **5h** displayed very good activity against all Aspergilus species, except for *A. fumigatus*, and Penicillium species, except for *P.v.c*, with MIC at 1.95 µmol/mL × 10^−2^ and MFC at 3.55 µmol/mL × 10^−2^, as well as against *T. viride* (MIC/MFC at 0.97/1.95 µmol/mL × 10^−2^). Compound **5f** showed good activity against all fungal species except for *A. fumigatus* with MIC/MFC at 2.09/3.92 µmol/mL × 10^−2^, while compound **5d** showed very good activity against *A. ochraceus* and *T. viride* with MIC at 1.88 µmol/mL × 10^−2^ and MFC at 3.52 µmol/mL × 10^−2^.

In general, most compounds showed good activity against *A. ochraceus* and *T. viride.* Thus, compounds **5a**–**5d**, **5f**–**5i** and **5k** exhibited excellent activity against *T. viride* with MIC in range of 0.97–2.12 µmol/mL × 10^−2^, while some of these compounds (**5b**–**5d, 5f**–**5i**, **5k**) showed good activity also against *A. ochraceus* with MIC in range of 1.88–2.10 µmol/mL × 10^−2^. Four compounds **5g**, **5k**, **5l** (MIC at 1.88–3.78 µmol/mL × 10^−2^) as well as **5i** (MIC 3.94 µmol/mL × 10^−2^) showed good activity against the most resistant to compounds tested fungal *A. fumigatus*.

Our results indicate that the described compounds are 13–52 times more potent than ketoconazole and 6- to 17-fold more potent than bifonazole.

According to the study of structure–activity relationships, it seems that the presence of 3-COOH, 3-OH groups on the benzene ring of indole derivatives (**5k**) is beneficial for antifungal activity, whereas attachment of the 6-methoxy group to the indole ring of compound **5k** results in the less potent derivative **5l**.

The order of activity of indole derivatives can be presented as **5k** > **5g** > **5a** > **5i** > **5e**. Thus, the combination of 3-COOH, 4-OH groups attached to benzene ring (**5k**) leads to the increase in activity of indole derivatives, while the opposite (**5a**) is less favorable. Additionally, the 3-COOH group on the benzene ring produced a positive effect on\ antifungal activity of indole derivatives. In the case of 5-methoxy indole derivatives, the 4-OH substitution (**5f**) was the most favorable, while 3-COOH (**5h**) and 3-OH substitution (**5c**) were much less favorable. Concerning the 6-methoxy indole derivatives: the combination of 3-COOH with 4-OH substituents in the benzene ring is favorable, whereas the combination of 3-OH and 4-COOH produced a negative effect on antifungal activity. Thus, the antifungal activity as, in case of antibacterial compounds, depends not only on the nature of substituents, but also on their relative positions.

Our results indicate that antibacterial and antifungal activities have different relationships with the chemical structure: the compound that showed the best antibacterial activity (**5b**) appeared to be one of the least active as antifungals. On the other hand, compounds **5g**, **5d** and **5e** expressed almost the same behavior against bacteria and fungi.

### 2.5. Docking Studies

#### 2.5.1. Docking to Antibacterial Targets

In order to elucidate the probable mechanism of action of designed compound docking studies to several antibacterial targets, such as *E. coli* DNA Gyrase, *S. aureus* Thymidylate kinase, *E. coli* Primase and *E. coli* MurA and MurB, were performed. The docking studies showed that the assessments of binding free energy to *E. coli* DNA Gyrase, Thymidylate kinase, *E. coli* Primase and *E. coli* MurA were higher than that to *E. coli* MurB; therefore, it may be resolved that the inhibition of *E. coli* MurB enzyme is the most probable, among the considered mechanism of action of the compounds, where binding scores were consistent with biological activity (Table 10).

**Figure 8:**
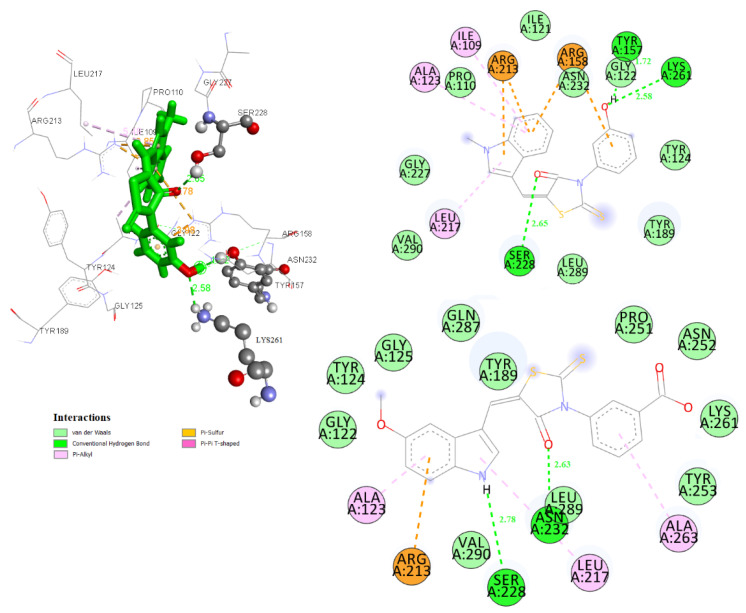
(**left**) Docked conformation of the most active compound **5b** in *E. coli* MurB. (**Right**) 2D diagrams of the most active compounds **5b** (up) and **5h** (down) in *E. coli* MurB.

**Figure 9:**
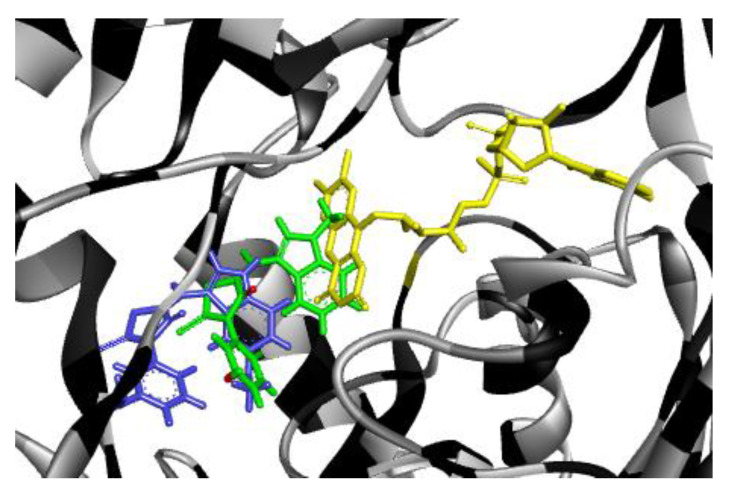
Docked conformation of compounds **5h** (green), **5b** (blue) and FAD (yellow) in *E. coli* MurB.

The most active compound **5b** in *E. coli* MurB enzyme showed three favorable hydrogen bond interactions. Two hydrogen bonds were observed between the hydroxyl substituent of the benzene ring of the compound and the residues Tyr157 and Lys261 (distance 1.72 Å and 2.58 Å, respectively), and another hydrogen bond interaction between the O atom of the carbonic group of the thiazolidine ring of the compound and Ser228 (distance 2.65 Å). The benzene ring interacts hydrophobically with the residues Tyr124, Gly122, Asn232 and Arg158, while the thizolidine ring interacts hydrophobically with the residues Tyr189 and Leu289 (Figure 8 and Figure 9). These interactions stabilize the complex compound enzyme and play a crucial role in the increased inhibitory action of the compound **5b**. Moreover, the hydrogen bond formation with the residue Ser228 is crucial for the inhibitory action of this compound, because this residue takes part in the proton transfer at the second stage of peptidoglycan synthesis [41]. Hydrogen bond interactions with the residue Ser228 were also observed for the rest of the studied compounds.

#### 2.5.2. Docking to Antifungal Targets

All the synthesized compounds and the reference drug ketoconazole were docked to lanosterol 14a-demethylase of *C. albicans* and DNA topoisomerase IV (Table 11).

**Figure 10:**
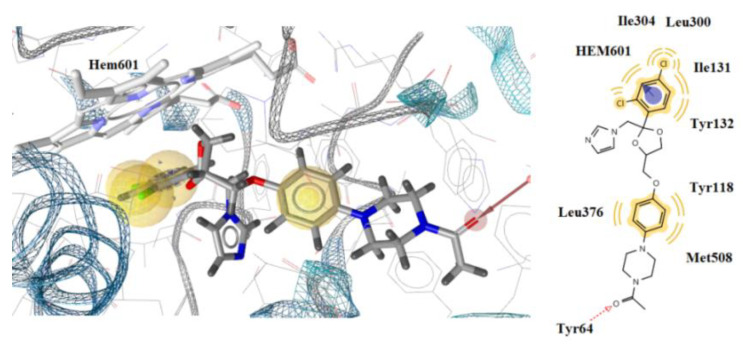
Docked conformation of ketoconazole in lanosterol 14alpha-demethylase of *C*. *albicans* (CYP51_ca_).

**Figure 11:**
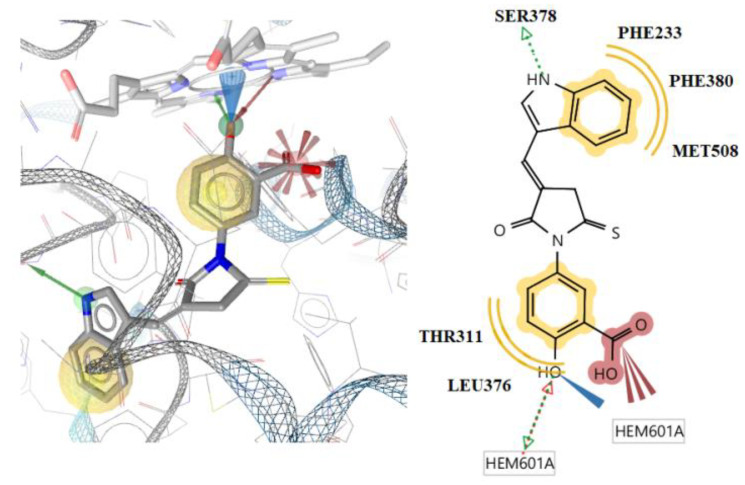
Docked conformation of compound **5k** in lanosterol 14alpha-demethylase of *C*. *albicans* (CYP51_ca_).

Docking results showed that the most active compound **5k** take place inside the active site of the enzyme interacting with the heme group of CYP51_Ca_ throughout its –COOH substituent of the benzene ring forming negative ionizable interactions with the heme group. Furthermore, the oxygen of the –OH substituent interacts with the Fe iron of the heme group and with the N atom of Heme, forming a hydrogen bond. Another hydrogen bond is formed between the N atom of indole moiety and Ser378. Hydrophobic interactions were also detected with the residues Thr311, Ley376, Phe233, Phe380 and Met308. Interaction with the heme group was also observed with the benzene ring of ketoconazole, which forms hydrophobic and aromatic interactions (Figure 10 and Figure 11). However, compound **5k** forms more and stronger interactions than ketoconazole and a more stable complex of the ligand with the enzyme. This is probably the reason why compound **5k** has better antifungal activity than ketoconazole.

### 2.6. Cytotoxicity Assessment

**Figure 12:**
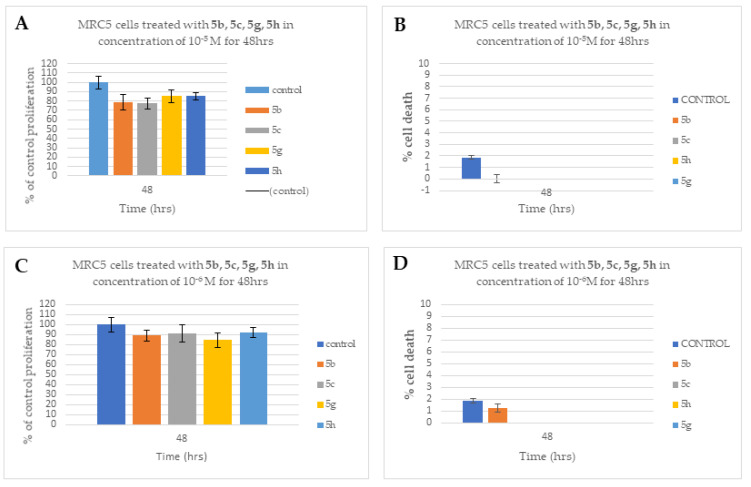
Assessment of cell proliferation of MRC-5 cells exposed to different compounds in culture. MRC-5 cells grown in culture were separately incubated with each of the compounds at concentrations 1 × 10^−5^ M (10μΜ) (panels **A**,**B**) and 1 × 10^−6^ M (1 μM) (panels **C**,**D**) for 48 h. The cell number was measured in cultures under the microscope using the Neubauer counting chamber, as indicated in “Methods” and expressed as % of the proliferation of control–untreated cultures (panels **A**,**C**). Moreover, the evaluation of cell death was assessed by the trypan-blue exclusion-dye assay, as shown in “Methods” (panels **B**,**D**). The results shown above indicate the mean numbers ± SD of two independent biological experiments (*n* ≥ 3). The diagrams shown above and the *t*-test statistical analysis were carried out using the GraphPad Prism 6.0 program. Notably, no statistical significance between the control–untreated culture with each one of treated compounds was obtained.

The assessment of cellular cytotoxicity of the compounds in normal human MRC-5 cells was evaluated at two concentrations in culture, i.e., 1 × 10^−5^ M (Figure 12A,B) and 1 × 10^−6^ M (Figure 12B,C). No substantial effect on cell proliferation after 48 h exposure has been observed in cultures, since the growth was ≥80% for all the tested agents compared to control untreated cultures (Figure 12A,C). Moreover, the percentage of dead cells accumulated in cultures was very low, since the maximum number did not exceed that of 2–2.5% (Figure 12B,D).

## 3. Materials and Methods

### 3.1. Chemistry

#### 3.1.1. General Procedure for the Synthesis 3-Aryl-2-thioxothiazolidin-4-ones **3a**–**e**

The mixture of bis (carboxymethyl) trithiocarbonate, (0.2 mol), of aromatic amine (0.2 mol) and 100 mL of the solvent was boiled for 5–8 h, cooled, the precipitate is filtered off, washed successively with diluted alcohol 1: 2, water, dried and recrystallized.

*3-(3-Hydroxyphenyl)-2-thioxothiazolidin-4-one* (**3a**).Yield 70%; m.p. 194–195 °C (AcOH). IR (cm^−1^): 3362.73 (OH), 1723.31 (C=O), 1596.98 (C=S). ^1^H-NMR (400 MHz, DMSO-*d*_6_, ppm) δ 9.79 (s, 1H, OH), 7.29 (t, *J* = 8.0 Hz, 1H, C_6_H_4_), 6.86 (ddd, *J* = 8.2, 2.3, 1.0 Hz, 1H, C_6_H_4_), 6.67–6.62 (m, 2H, C_6_H_4_), 4.36 (s, 2H, CH_2_). ^13^C NMR (101 MHz, dmso) δ 203.50, 173.94, 157.94, 136.39, 129.87, 119.04, 116.20, 115.59, 37.01. Anal. Calcd. for C_9_H_7_NO_2_S_2_ (%): C 47.98; H, 3.13; N, 6.22; S, 28.46. Found (%):C 48.06; H, 3.04; N, 6.31; S, 28.54.

3*-(4-Hydroxyphenyl)-2-thioxothiazolidin-4-one* (**3b**). Yield 85%. m.p. 250–252 °C (AcOH). IR (cm^−1^): 3442.77 (OH), 1742.6 (C=O), 1596.02 (C=S). ^1^H-NMR (400 MHz, DMSO-*d*_6_, ppm) δ 9.82 (s, 1H, OH), 7.05–6.99 (m, 2H, C_6_H_4_), 6.88–6.83 (m, 2H, C_6_H_4_), 4.34 (s, 2H, CH_2_). ^13^C NMR (101 MHz, dmso) δ 203.93, 174.16, 157.90, 129.67, 126.39, 115.69, 36.69. Anal. Calcd. for C_9_H_7_NO_2_S_2_ (%): C 47.98; H, 3.13; N, 6.22; S, 28.46. Found (%):C 47.89; H, 3.06; N, 6.15; S, 28.41.

*3-(4-Oxo-2-thioxothiazolidin-3-yl)benzoic acid* (**3c**). Yield 83%. m.p. 252–254 °C (AcOH-DMFA). IR (cm^−1^): 1740.67 (C=O), 1688.6 (C=O), 1586.38. (C=S). ^1^H-NMR (400 MHz, DMSO-*d*_6_, ppm) δ 13.24 (s, 1H, COOH), 8.06–8.01 (m, 1H, C_6_H_4_), 7.87 (t, *J* = 1.7 Hz, 1H, C_6_H_4_), 7.66 (t, *J* = 7.8 Hz, 1H, C_6_H_4_), 7.54 (ddd, *J* = 7.9, 2.1, 1.2 Hz, 1H, C_6_H_4_), 4.37 (s, 2H, CH_2_). ^13^C NMR (101 MHz, dmso) δ 203.72, 174.00, 166.37, 135.84, 133.29, 131.96, 129.97, 129.76, 129.60, 37.28. Anal. Calcd. for C_10_H_7_NO_3_S_2_ (%): C 47.42; H, 2.79; N, 5.53; S, 25.32. Found (%): C 47.54; H, 2.68; N, 5.41; S, 25.41.

*4-(4-Oxo-2-thioxothiazolidin-3-yl)benzoic acid* (**3d**). Yield 89%. m.p. >270 °C (AcOH-DMFA). IR (cm^−1^): 1722.35 (C=O), 1697.28 (C=O), 1503.44. (C=S). ^1^H-NMR (400 MHz, DMSO-*d*_6_, ppm) δ 13.12 (s, 1H, COOH), 8.10–8.05 (m, 2H, C_6_H_4_), 7.45–7.39 (m, 2H, C_6_H_4_), 4.40 (s, 1H, CH_2_). ^13^C NMR (101 MHz, dmso) δ 203.35, 173.81, 166.55, 139.30, 131.53, 130.16, 129.11, 37.29. ^13^C NMR (101 MHz, dmso) δ 203.95, 174.06, 171.02, 161.26, 135.86 (s), 130.69, 126.52, 117.93, 113.50, 36.99.Anal. Calcd. for C_10_H_7_NO_3_S_2_ (%): C 47.42; H, 2.79; N, 5.53; S, 25.32. Found (%): C 47.36; H, 2.71; N, 5.62; S, 25.22.

*2-Hydroxy-5-(4-oxo-2-thioxo-thiazolidin-3-yl)-benzoic acid* (**3e**). Yield 61%. m.p. >270 °C (AcOH-DMFA). IR (cm^−1^): 1748.39 (C=O), 1672.2 (C=O), 1486.08. (C=S). ^1^H-NMR (400 MHz, DMSO-*d*_6_, ppm) δ 7.71 (d, *J* = 2.6 Hz, 1H, C_6_H_3_), 7.39 (dd, *J* = 8.8, 2.6 Hz, 1H, C_6_H_3_), 7.09 (d, *J* = 8.8 Hz, 1H, C_6_H_3_), 4.33 (s, 2H, CH_2_). Anal. Calcd. for C_10_H_7_NO_4_S_2_ (%): C 44.60; H, 2.62; N, 5.20; S, 23.81. Found (%): C C 44.73; H, 2.55; N, 5.13; S, 23.90.

#### 3.1.2. General Procedure for the Synthesis 3-Aryl-5-(1*H*-indol-3-ylmethylene)-2-thioxothiazolidin-4-ones 5**a**–**l**

A mixture of 3-aryl-2-thioxothiazolidin-4-one **3a**–**e** (5 mmol), the corresponding 1*H*-indole-3-carbaldehyde **4a**–**d** (6 mmol) and ammonium acetate (5 mmol, 0.39g) in acetic acid (10 mL) was heated to boiling for 2 h, cooled, the precipitate is filtered off, washed successively with acetic acid, alcohol and water, dried and recrystallized with AcOH-DMFA mixture.

*(Z)-3-(3-Hydroxyphenyl)-5-(1H-indol-3-ylmethylene)-2-thioxothiazolidin-4-one* (**5a**). Yield 78%; m.p. 271–273 °C. ^1^H-NMR (400 MHz, DMSO-*d*_6_, ppm) δ 12.38 (s, 1H, NH), 9.83 (s, 1H, OH), 8.09 (s, 1H, CH = ), 7.97 (dd, *J* = 9.3, 5.2 Hz, 2H, H_4_ +H_7_, indole), 7.53 (d, *J* = 7.8 Hz, 1H, H_2_ indole), 7.37–7.19 (m, 3H, C_6_H_4_ + H_5_ +H_6_, indole), 6.90 (ddd, *J* = 8.3, 2.4, 0.9 Hz, 1H, C_6_H_4_), 6.82–6.73 (m, 2H, C_6_H_4_). ^13^C-NMR (101 MHz, DMSO-*d*_6_, ppm) δ 192.73, 166.58, 157.99, 136.45, 136.39, 130.46, 129.90, 126.75, 125.86, 123.34, 121.48, 119.13, 118.54, 116.27, 115.68, 115.28, 112.55, 111.11. MS (ESI): *m*/*z* = 353.0 [M + H]^+^. Anal. Calcd. for C_18_H_12_N_2_O_2_S_2_ (%): C, 61.34; H, 3.43; N, 7.95; S, 18.20. Found (%): C, 61.45; H, 3.36; N, 7.86; S, 18.29.

*(Z)-3-(3-Hydroxyphenyl)-5-(1-methyl-1H-indol-3-ylmethylene)-2-thioxothiazolidin-4-one* (**5b**). Yield 92%; m.p. >275 °C. IR (cm^−1^): 3356.94 (OH), 1707.88 (C=O), 1670.27 (C=O), 1595.05 (C=C), 1572.87 (C=S). ^1^H-NMR (300 MHz, DMSO-*d*_6_, ppm) δ 9.49 (s, 1H, OH), 8.02 (s, 1H, CH = ), 7.89 (d, *J* = 7.6 Hz, 1H, H_4_ indole), 7.85 (s, 1H, H_7_ indole), 7.50 (d, *J* = 7.9 Hz, 1H, H_2_ indole), 7.36–7.20 (m, 3H, C_6_H_4_ + H_5_ +H_6_, indole), 6.94–6.88 (m, 1H, C_6_H_4_), 6.72–6.66 (m, 2H, C_6_H_4_), 3.99 (s, 3H, CH_3_N). ^13^C-NMR (101 MHz, DMSO-*d*_6_, ppm) δ 192.66, 166.54, 157.98, 136.92, 136.42, 133.76, 129.92, 127.24, 125.17, 123.39, 121.80, 119.12, 118.62, 116.26, 115.65, 114.98, 110.90, 110.09, 33.38. MS (ESI): *m*/*z* = 367.2 [M + H]^+^. Anal. Calcd. for C_19_H_14_N_2_O_2_S_2_ (%): C, 62.27; H, 3.85; N, 7.64; S, 17.50. Found (%):C, 62.34; H, 3.77; N, 7.59; S, 17.41.

*(Z)-3-(3-Hydroxyphenyl)-5-(5-methoxy-1H-indol-3-ylmethylene)-2-thioxothiazolidin-4-one* (**5c**). Yield 82%; m.p. 268–270°C. IR (cm^−1^): 3247.01 (OH), 3263.4 (NH) 1676.06 (C=O), 1591.2 (C=C), 1572.87 (C=S) ^1^H-NMR (300 MHz, DMSO-*d*_6_, ppm) δ 12.08 (s, 1H, NH), 9.55 (s, 1H, OH), 8.07 (s, 1H, CH = ), 7.68 (d*, J* = 2.4 Hz, 1H, H_4_ indole), 7.40–7.25 (m, 3H, C_6_H_4_ + H_2_ +H_7_, indole), 6.89 (d, *J* = 7.9 Hz, 1H, C_6_H_4_), 6.82 (d, *J* = 9.5 Hz, 1H, H_6_ indole), 6.70–6.61 (m, 2H, C_6_H_4_), 3.86 (s, 3H, CH_3_O). ^13^C-NMR (101 MHz, DMSO-*d*_6_, ppm) δ 192.68, 166.53, 157.99, 155.27, 136.51, 131.16, 130.50, 129.91, 127.76, 126.45, 119.13, 116.24, 115.68, 114.40, 113.55, 113.33, 111.22, 100.37, 55.48. MS (ESI): *m*/*z* = 383.2 [M + H]^+^. Anal. Calcd. for C_19_H_14_N_2_O_3_S_2_ (%): C, 59.67; H, 3.69; N, 7.32; S, 16.77. Found (%): C, 59.51; H, 3.74; N, 7.26; S, 16.85.

*(Z)-3-(3-Hydroxyphenyl)-5-(6-methoxy-1H-indol-3-ylmethylene)-2-thioxothiazolidin-4-one* (**5d**). Yield 87%; m.p. 272–274 °C. IR (cm^−1^): 3343.44 (OH), 3263.4 (NH) 1683.78 (C=O), 1592.16 (C=C), 1570.95 (C=S) ^1^H-NMR (300 MHz, DMSO-*d*_6_, ^1^H-NMR (300 MHz, DMSO-*d*_6_, ppm) δ 11.97 (s, 1H, NH), 9.55 (s, 1H, OH), 7.99 (s, 1H, CH = ), 7.72 (d, J = 8.6 Hz, 1H, H_4_ indole), 7.61 (s, 1H, H_2_ indole), 7.29 (t, J = 8.1 Hz, 1H, C_6_H_4_), 6.95 (s, 1H, H_7_ indole), 6.89 (d, *J =* 8.0 Hz, 1H, C_6_H_4_), 6.81 (d, *J* = 8.2 Hz, 1H, H_5_ indole), 6.66 (s, 2H, C_6_H_4_), 3.84 (s, 3H, CH_3_O). ^13^C-NMR (101 MHz, DMSO-*d*_6_, ppm) δ 192.66, 166.55, 157.98, 156.84, 137.31, 136.46, 129.90, 129.55, 126.11, 120.68, 119.33, 119.12, 116.26, 115.66, 115.06, 111.44, 111.28, 95.36, 55.27. MS (ESI): *m*/*z* = 383.2 [M + H]^+^. Anal. Calcd. for C_19_H_14_N_2_O_3_S_2_ (%): C, 59.67; H, 3.69; N, 7.32; S, 16.77. Found (%): C, 59.75; H, 3.76; N, 7.27; S, 16.64.

*(Z)-3-(4-Hydroxyphenyl)-5-(1H-indol-3-ylmethylene)-2-thioxothiazolidin-4-one* (**5e**). Yield 94%; m.p. >275 °C. IR (cm^−1^): 3382.98 (OH, NH) 1679.92 (C=O), 1592.16 (C=C), 1574.8 (C=S) ^1^H-NMR (400 MHz, DMSO-*d*_6_, ppm) δ 12.37 (s, 1H, NH), 9.86 (s, 1H, OH), 8.09 (s, 1H, CH = ), 8.00–7.92 (m, 2H, H_4_ +H_7_, indole), 7.53 (d, *J* = 8.0 Hz, 1H, H_2_ indole), 7.32–7.19 (m, 2H, H_5_ +H_6_, indole), 7.16 (d, *J* = 8.8 Hz, 2H, C_6_H_4_), 6.89 (d, *J* = 8.8 Hz, 2H, C_6_H_4_). ^13^C-NMR (101 MHz, DMSO-*d*_6_, ppm) δ 193.15, 166.78, 157.98, 136.38, 130.39, 129.76, 126.75, 126.43, 125.77, 123.32, 121.46, 118.51, 115.70, 115.19, 112.54, 111.10. MS (ESI): *m*/*z* = 353.2 [M + H]^+^. Anal. Calcd. for C_18_H_12_N_2_O_2_S_2_ (%): C, 61.34; H, 3.43; N, 7.95; S, 18.20. Found (%): C, 61.30; H, 3.29; N, 7.99; S, 18.12.

*(Z)-3-(4-Hydroxyphenyl)-5-(5-methoxy-1H-indol-3-ylmethylene)-2-thioxothiazolidin-4-one* (**5f**). Yield 88%; m.p. 264–266 °C. IR (cm^−1^): 3250.86 (OH, NH) 1669.31(C=O), 1570.95 (C=C), 1510.19 (C=S). ^1^H-NMR (300 MHz, DMSO-*d*_6_, ppm) δ 12.25 (s, 1H, NH), 9.87 (s, 1H, OH), 8.14 (s, 1H, CH = ), 7.87 (d, *J* = 2.9 Hz, 1H, H_4_ indole), 7.52 (s, 1H, H_2_ indole), 7.40 (d, *J* = 8.8 Hz, 1H, H_7_ indole), 7.15 (d, J = 8.6 Hz, 2H, C_6_H_4_), 6.92–6.86 (m, 3H, C_6_H_4_ + H_6_ indole), 3.83 (s, 3H, CH_3_O). ^13^C-NMR (101 MHz, DMSO-*d*_6_, ppm) δ 193.08, 166.75, 157.94, 155.25, 131.12, 130.39, 129.74, 127.73, 126.48, 126.33, 115.72, 114.30, 113.51, 113.33, 111.20, 100.29, 55.45. MS (ESI): *m*/*z* = 383.2 [M + H]^+^. Anal. Calcd. for C_19_H_14_N_2_O_3_S_2_ (%): C, 59.67; H, 3.69; N, 7.32; S, 16.77. Found (%): C, 59.71; H, 3.61; N, 7.24; S, 16.83.

*(Z)-3-[5-(1H-Indol-3-ylmethylene)-4-oxo-2-thioxo-thiazolidin-3-yl]-benzoic acid* (**5g**). Yield 82%; m.p. >275 °C. IR (cm^−1^): 3230.61 (OH, NH), 1722.35 (C=O), 1693.42 (C=O), 1596.02 (C=C), 1577.7 (C=S).^1^H-NMR (300 MHz, DMSO-*d*_6_, ppm) δ 12.73 (s, COOH), 12.25 (s, NH), 8.15–8.06 (m, 2H, C_6_H_4_ + CH = ), 7.96–7.84 (m, 2H, C_6_H_4 +_ H_4_ indole), 7.78 (d, *J* = 2.9 Hz, 1H, H_7_ indole), 7.66 (t, *J* = 7.8 Hz, 1H, C_6_H_4_), 7.56–7.46 (m, 2H, C_6_H_4_ + H_2_ indole), 7.28–7.14 (m, 2H, H_5_ +H_6_, indole). ^13^C NMR (101 MHz, dmso) δ 192.94, 166.54, 136.41, 135.85, 133.28, 132.18, 130.56, 130.02, 129.83, 129.58, 126.75, 126.03, 123.36, 121.51, 118.52, 115.32, 112.57, 111.09. Anal. Calcd. for C_19_H_12_N_2_O_3_S_2_ (%): C, 59.99; H, 3.18; N, 7.36; S, 16.86. Found (%):C, 60.08; H, 3.09; N, 7.45; S, 16.92.

*(Z)-3-[5-(5-Methoxy-1H-indol-3-ylmethylene)-4-oxo-2-thioxothiazolidin-3-yl]-benzoic acid* (**5h**). Yield 72%; m.p. >275 °C. IR (cm^−1^): 3248.93 (OH, NH), 1706.92 (C=O), 1587.34 (C=C), 1575.77 (C=S).^1^H-NMR (400 MHz, DMSO-*d*_6_, ppm) δ δ 13.22 (s, 1H, COOH), 12.29 (s, 1H, NH), 8.17 (s, 1H, CH = ), 8.07 (dd, *J* = 6.3, 2.5 Hz, 1H, C_6_H_4_), 7.98 (s, 1H, H_4_ indole), 7.90 (d, *J* = 2.8 Hz, 1H, C_6_H_4_), 7.73–7.66 (m, 2H, C_6_H_4_ + H_7_ indole), 7.54 (d, J = 2.0 Hz, 1H, C_6_H_4_), 7.40 (t, *J* = 7.4 Hz, 1H, H_2_ indole), 6.89 (dd, *J* = 8.7, 2.3 Hz, 1H, H_6_ indole), 3.83 (s, 3H, CH_3_O). ^13^C-NMR (101 MHz, DMSO-*d*_6_, ppm) δ 192.89, 166.49, 155.30, 135.92, 133.36, 132.00, 131.16, 130.57, 130.01, 129.84, 129.62, 127.76, 126.62, 114.44, 113.54, 113.34, 111.21, 100.38, 55.48. MS (ESI): *m*/*z* = 411.2 [M + H]^+^. Anal. Calcd. C_20_H_14_N_2_O_4_S_2_ (%): C, 58.52; H 3.44; N, 6.82; S, 15.62. Found (%):C, 58.65; H 3.33; N, 6.76; S, 15.71.

*(Z)-4-[5-(1H-Indol-3-ylmethylene)-4-oxo-2-thioxothiazolidin-3-yl]-benzoic acid* (**5i**). Yield 82%; m.p. >275 °C. IR (cm^−1^) 3433.13 (OH), 3228.68 (NH), 1712.71 (C=O), 1689.56 (C=O), 1596.98 (C=C), 1574.8 (C=S).^1^H-NMR (300 MHz, DMSO-*d*_6_, ppm) δ 12.90 (s, COOH), 12.25 (s, NH), 8.15 (d, *J* = 8.4 Hz, 2H, C_6_H_4_), 8.09 (s, 1H, CH = ), 7.88 (d, *J* = 7.2 Hz, 1H, H_4_ indole), 7.78 (d, *J* = 3.0 Hz, 1H, H_7_ indole), 7.49 (d, *J* = 7.3 Hz, 1H, H_2_ indole), 7.42 (d, *J* = 8.4 Hz, 2H, C_6_H_4_), 7.28–7.13 (m, 2H, H_5_ +H_6_, indole). ^13^C-NMR (101 MHz, DMSO-*d*_6_, ppm) δ 192.61, 166.53, 139.38, 136.41, 131.58, 130.65, 130.15, 129.23, 126.76, 126.21, 123.38, 121.54, 118.54, 115.13, 112.58, 111.09. MS (ESI): *m*/*z* = 381.2 [M + H]^+^. Anal. Calcd. C_19_H_12_N_2_O_3_S2 (%): C, 59.99; H, 3.18; N, 7.36; S, 16.86. Found (%):C, 60.07; H, 3.09; N, 7.28; S, 16.95.

*(Z)-4-[5-(6-Methoxy-1H-indol-3-ylmethylene)-4-oxo-2-thioxothiazolidin-3-yl]-benzoic acid* (**5j**). Yield 84%; m.p. >275 °C. IR (cm^−1^) 3254.72 (OH), 1704.99 (C=O), 1691.49 (C=O), 1596.98 (C=C), 1576.73 (C=S).^1^H-NMR (300 MHz, DMSO-*d*_6_, ppm) δ 12.59 (s, 1H, COOH), 12.04 (s, 1H, NH), 8.14 (d, *J* = 5.6 Hz, 2H, C6H4), 8.02 (s, 1H, CH = ), 7.73 (d, *J* = 7.7 Hz, 1H, H_4_ indole), 7.65 (s, 1H, H_2_ indole), 7.42 (d, *J* = 6.6 Hz, 2H, C_6_H_4_), 6.95 (s, 1H, H_7_ indole), 6.82 (d*, J* = 6.1 Hz, 1H, H_5_ indole), 3.83 (s, 3H, CH_3_O). ^13^C-NMR (101 MHz, DMSO-*d*_6_, ppm) δ 192.54 (s), 166.51, 156.87, 139.39, 137.34, 131.54, 130.13, 129.75, 129.22, 126.46, 120.68, 119.35, 114.91, 111.49, 111.27, 95.38, 55.26. MS (ESI): *m*/*z* = 411.2 [M + H]^+^. Anal. Calcd. C_20_H_14_N_2_O_4_S_2_ (%): C, 58.52; H 3.44; N, 6.82; S, 15.62. Found (%):C, 58.47; H 3.49; N, 6.79; S, 15.54.

*(Z)-2-Hydroxy-5-[5-(1H-indol-3-ylmethylene)-4-oxo-2-thioxothiazolidin-3-yl]-benzoic acid* (**5k**). Yield 93%; m.p. >275 °C. IR (cm^−1^) 3226.75 (OH, NH), 1718.49 (C=O), 1678.95 (C=O), 1597.95 (C=C), 1577.7 (C=S).^1^H-NMR (300 MHz, DMSO-*d*_6_, ppm) δ 12.24 (s, 1H, NH), 11.54 (s, 1H, COOH), 8.07 (s, 1H, CH = ), 7.87 (d, *J* = 7.4 Hz, 1H, H_4_ indole), 7.76 (s, 2H, C_6_H_3_ + H_7_ indole), 7.49 (d, *J* = 7.5 Hz, 1H, H_2_ indole), 7.39 (dd, *J* = 8.8, 2.4 Hz, 1H, C_6_H3), 7.25–7.16 (m, 2H, H_5_ +H_6_, indole), 7.06 (d, *J* = 8.8 Hz, 1H, C_6_H_3_). ^13^C-NMR (101 MHz, DMSO-*d*_6_, ppm) δ 193.19, 171.09, 166.68, 161.33, 136.37, 135.92, 130.76, 130.44, 126.73, 126.51, 125.89, 123.36, 121.51, 118.47, 117.94, 115.28, 113.54, 112.56, 111.07. MS (ESI): *m*/*z* = 397.0 [M + H]^+^. Anal. Calcd. C_19_H_12_N_2_O_4_S_2_ (%): C, 57.56; H 3.05; N, 7.07; S, 16.18. Found (%):C, 57.64; H 3.12; N, 7.01; S, 16.25.

*(Z)-2-Hydroxy-5-[5-(6-methoxy-1H-indol-3-ylmethylene)-4-oxo-2-thioxothiazolidin-3-yl]-benzoic acid* (**5l**). Yield 72%; m.p. >275 °C. IR (cm^−1^) 3247.97 (OH, NH), 1717.53 (C=O), 1677.03 (C=O), 1599.88 (C=C), 1576.73 (C=S).^1^H-NMR (300 MHz, DMSO-*d*_6_, ppm) δ 12.00 (s, NH), 11.52 (s, 1H, COOH), 8.00 (s, CH = ), 7.79–7.69 (m, 2H, C_6_H_3_, H_4_ indole), 7.62 (d, *J* = 2.1 Hz, 1H, H_2_ indole), 7.37 (dd, *J* = 8.8, 2.2 Hz, 1H, C_6_H_3_), 7.05 (d, *J* = 8.8 Hz, 1H, C_6_H_3_), 6.94 (s, 1H, H_7_ indole), 6.81 (d, *J* = 8.7 Hz, 1H, H_5_ indole), 3.84 (s, 3H, CH_3_O). ^13^C-NMR (101 MHz, DMSO-*d*_6_, ppm) δ 193.15, 171.08, 166.65, 161.34, 156.84, 137.30, 135.88, 130.74, 129.55, 126.49, 126.12, 120.67, 119.29, 117.91, 115.08, 113.61, 111.46, 111.24, 95.36, 55.26. MS (ESI): *m*/*z* = 427.0 [M + H]^+^. Anal. Calcd. C_20_H_14_N_2_O_5_S_2_ (%): C, 56.33; H 3.31; N, 6.57; S, 15.04. Found (%):C, 56.26; H 3.28; N, 6.66; S, 14.95.

### 3.2. PASS and PASS-Based Web Applications

PASS predictions are based on the structure–activity relationships derived from the data on over eight thousand biological activities of more than one million molecules included in the training set [37,42,43,44]. Structure–activity relationships are examined using MNA (Multilevel Neighborhoods of Atoms) structural descriptors [45] and modified the Naïve Bayes approach [46]. Structural formulae presented as MDL MOL or SDF files [47] are used as input information. PASS output is the list of predicted activities with two assessments: Pa is the estimate of the probability of belonging to the class of active compounds, and Pi is the estimate of the probability of belonging to the class of inactive ones [46]. The higher the probability difference Pa-Pi, the higher the chance to confirm the prediction in the following experiment.

Since 1999 [48], the PASS Online web application has been freely available via the Internet. It provides an opportunity to predict several thousand biological activities with an average probability of about 95% [39]. Comparing PASS Online with some other freely available web services predicting biological activity profiles demonstrated its superiority in performance [49]. Using the special training sets created based on ChEMBL data [50] we developed several specialized PASS-based web applications: AntiBac-Pred [38,51], AntiFun-Pred [52], KinScreen [53,54], which predict the detailed antibacterial, antifungal and kinase inhibitory activity profiles, respectively. These web applications differ from the standard PASS version only by the training sets focused on the particular pharmacotherapeutic fields. Therefore, the interpretation of the prediction results is the same as described above.

### 3.3. Ligand-Based Pharmacophore Modeling

The LigandScout program (Advanced version 4.4.7) [55] with default settings was used to perform the pharmacophore modeling studies. Prior to the generation of pharmacophore hypotheses, all tested dataset compounds were built using ChemDraw Ultra (CambridgeSoft, version 12.0) and converted into the 3D format. The lowest energy conformations were generated using *MMFF94* (Merck molecular force field) and the BEST conformation model generation method was used during conformer generation with a maximum number of 250 conformations, an energy threshold value of 20 kcal/mol above the global energy minimum and an RMS threshold of 0.8.

Compounds **1–9** were selected for training. The training set molecules play a key role in determining the quality of the pharmacophore models generated, while the test set compounds serve to validate the resultant pharmacophore solutions.

Based on the feature mapping results, five matching features were selected, including hydrophobic features (H, yellow), aromatic rings (AR, blue), hydrogen bond donors (HBD, green), hydrogen bond acceptors (HBA, red) and negative ionizable features (red star). The quality of generated hypotheses was ranked based on the pharmacophore fit score, which indicates the modality of the mapping between a molecule and a model. A value of 1 reflects the best prediction [56]. The highest rank score hypotheses for antibacterial activity were considered statistically the best hypotheses and selected for the further analysis.

#### 3.3.1. Pharmacophore Validation

The generated pharmacophore hypothesis was validated using a test set and leave-one-out methods.

##### Pharmacophore Validation Using Test Set

The test set method is used to clarify whether the generated pharmacophore model is capable to predict the antibacterial activity of compounds other than the training set compounds and categorize them properly in their activity scale. For the test set, compounds **10**–**14** were selected. For the test set compounds, the conformation generation was performed using default values and BEST conformation analysis algorithms [57,58].

##### Pharmacophore Validation Using Leave-One-Out

The pharmacophore model was cross-validated by the leave-one-out method. In this method, pharmacophore models are recomputing again by leaving one compound at a time from the training set compounds, until each compound was left out once, and its affinity is predicted using that new model [59]. This validation is performed to verify that the correlation of the original pharmacophore model does not depend only on one particular compound [57,60]. By leaving each one of the 9 training set compounds, 9 new models were generated. Thus, we did not obtain any meaningful differences between Model-1 and each model generated from this method, validating our pharmacophore model.

### 3.4. Biological Evaluation

#### 3.4.1. Antibacterial Action

The following Gram-negative bacteria: *Escherichia coli* (ATCC 35210), *Enterobacter cloacae* (*clinical isolate*), *Salmonella* Typhimurium (ATCC 13311), *Pseudomonas aeruginosa* (ATCC 27853) as well as Gram-positive bacteria: *Listeria monocytogenes* (NCTC 7973), *Bacillus cereus* (clinical isolate), *Micrococcus luteus* (ATCC 10240) and *Staphylococcus aureus* (ATCC 6538) were used. The organisms were obtained from the Mycological Laboratory, Department of Plant Physiology, Institute for Biological Research “Siniša Stankovic”-National Institute of Republic of Serbia, Belgrade, Serbia.

The minimum inhibitory (MIC) and minimum bactericidal (MBC) concentrations were determined by the modified microdilution method, as previously reported [34,60].

Resistant strains used in microdilution assay were isolates of *S. aureus* (strain isolated from cow), *E. coli* (strain isolated form pig) and *P. aeruginosa* (strain isolated from cat) obtained as described in Kartsev et al. [61].

#### 3.4.2. Inhibition of Biofilm Formation

The method was performed as described [62] with some modifications. Briefly, the *P. aeruginosa* resistant strain was incubated with MIC and subMIC of tested compounds in Triptic soy broth enriched with 2% glucose at 37 °C for 24 h. After 24 h, each well was washed twice with sterile PBS (phosphate buffered saline, pH 7.4) and fixed with methanol for 10 min. Methanol was then removed and the plate was air dried. Biofilm was stained with 0.1% crystal violet (Bio-Merieux, Lyon, France) for 30 min. Wells were washed with water, air dried and 100 μL of 96% ethanol (Zorka, Serbia) was added. The absorbance was read at 620 nm on a Multiskan™ FC Microplate Photometer, Thermo Scientific™. The percentage of inhibition of biofilm formation was calculated by the formula:

[(A_620_ control − A_620_ sample)/A_620_ control] × 100.

#### 3.4.3. Checkboard Assay

It was carried out with 96-well microplates containing TSB medium for the resistant *P. aeruginosa* strain, supplemented with examined compounds in concentrations ranging from 1/16 to 4 × MIC as described previously [60] in the checkboard manner. The fractional inhibitory concentration index (FICI) was calculated by the following equation as described in our previous paper [63]:

FICI = FIC1^0^/MIC1^0^ + FIC2^0^/MIC2^0^

FIC1^0^ and FIC2^0^ are the MICs of a combination of tested compounds and antibiotics, and MIC1^0^ and MIC2^0^, represent the MIC values of individual agents. The following cut-offs: FIC ≤ 0.5 synergistic, >0.5 <2 additive, ≥2 <4 indifferent, and FIC > 4 antagonistic effects were used for the discussion of obtained results.

#### 3.4.4. Antifungal Activity

For the antifungal bioassays, six fungi were used: *Aspergillus niger* (ATCC 6275), *Aspergillus fumigatus* (ATCC 1022), *Aspergillus versicolor* (ATCC 11730), *Aspergillus ochraceus* (ATCC 12066), *Penicillium funiculosum* (ATCC 36839), *Trichoderma viride* (IAM 5061), *Penicillium verrucosum var. cyclopium* (food isolate), *Penicillium ochrochloron* (ATCC 9112). The organisms were obtained from the Mycological Laboratory, Department of Plant Physiology, Institute for Biological Research “Siniša Stankovic”, Belgrade, Serbia [64,65].

### 3.5. Docking Studies

Τhe AutoDock 4.2^®^ software was used for the docking stimulation. The free energy of binding (ΔG) of *E. coli* DNA GyrB, Thymidylate kinase, *E. coli* MurA, *E. coli* primase, *E. coli* MurB, DNA topoIV and CYP51 of *C*. *albicans* in complex with the inhibitors were generated using this molecular docking program. Regarding the X-ray crystal structures, data of all the enzymes used were obtained from the Protein Data Bank (PDB ID: 1KZN, AQGG, 1DDE, JV4T, 2Q85, 1S16 and 5V5Z, respectively). All procedures were performed according to our previous paper [66].

### 3.6. Assessment of Cytotoxicity

The normal human lung fibroblast MRC-5 cell line is stored and used in our laboratory in a routine manner (passage < 40). MRC-5 cells were grown in culture (37 °C, humidified atmosphere containing 5% *v/v* CO_2_) in DMEM medium supplemented with 10% *v*/*v* FBS, 1% PS penicillin–streptomycin). The compounds tested were dissolved in DMSO and stored in 4 °C. For the assessment of cytotoxicity, the cells were seeded in a 96-well plate at an initial concentration of 5 × 10^4^ cells/mL and allowed to attach for at least 3h before the addition of the compounds at two different concentrations: 1 × 10^−5^ M (10 μΜ) and 1 × 10^−6^ M (1 μΜ). Note that the concentration of DMSO in culture was ≤0.2% *v*/*v*, in which no detectable effect on cell proliferation is observed [67]. To assess the cytotoxicity of each compound, the cells were allowed to grow for additional 48 h before their number is estimated in culture using the Neubauer counting chamber under an optical microscope. Cell growth in each treated culture is expressed as the percentage compared to that seen for the untreated control cells. Moreover, the number of dead cells was also measured using the Trypan-blue method, as previously described [65,66,67]. Statistical t-test analysis was performed via the use of GraphPad Prism 6.0 program.

## 4. Conclusions

Twelve 3-aryl-5-(1*H*-indol-3-ylmethylene)-2-thioxothiazolidin-4-ones **5a**–**l** were designed, synthesized and evaluated in silico and experimentally for their antimicrobial actions against the panel of Gram positive and Gram negative bacteria and fungi.

It should be mentioned that all compounds appeared to be more potent than ampicillin against all bacteria tested and streptomycin against all bacteria except *B. cereus*, and *En. Cloacae*. The most sensitive bacteria were found to be *S. aureus*, while *L. monocytogenes* was the most resistant one. Compounds also appeared to be active against three resistant strains MRSA, *E. coli* and *P. aeruginosa*, showing better activity against MRSA than both reference drugs, while showing better activity against the other two resistant strains than ampicillin.

Concerning antifungal action, the tested compounds exhibited very good activity against all the fungal species tested, being more active than ketoconazole and bifonazole. The most sensitive fungal strain appeared to be *T. viride*, while the most resistant filamentous *A. fumigatus*.

It can be observed that the growth of both Gram-negative and Gram-positive bacteria and fungi responded differently to the tested compounds, which indicates that different substituents may lead to different modes of action or that the metabolism of some bacteria/fungi was better able to overcome the effect of the compounds or adapt to it.

Docking analysis to DNA Gyrase, Thymidylate kinase and *E. coli* MurB indicated a probable involvement of MurB inhibition in the antibacterial mechanism of compounds tested while docking analysis to 14α-lanosterol demethylase (CYP51) and tetrahydrofolate reductase of *Candida albicans* indicated a probable implication of CYP51 reductase at the antifungal activity of the compounds and secondary involvement of dihydrofolate reductase inhibition at the mechanism of action of the most active compounds.

Finally, compounds **5b** (Z)-3-(3-hydroxyphenyl)-5-((1-methyl-1*H*-indol-3-yl)methylene)-2-thioxothiazolidin-4-one Z)-3-[5-(1*H*-Indol-3-ylmethylene)-4-oxo-2-thioxo-thiazolidin-3-yl]-benzoic acid as well as **5h** (Z)-3-(5-((5-methoxy-1*H*-indol-3-yl)methylene)-4-oxo-2-thioxothiazolidin-3-yl)benzoic acid can be considered as lead compounds for further development of more potent and safe antibacterial and antifungal agents.

## Figures and Tables

**Table 1:** Formation of 3-aryl-2-thioxothiazolidin-4-ones **3a**–**e**.

No	Starting Amin	Reaction Product	Solvent	Reaction Time (Hours)	Yield, %
1	**1a**	**3a**	*i*-PrOH:H_2_O/1:2	8	72
2	**1b**	**3b**	*i*-PrOH:H_2_O/1:1	8	85
3	**1c**	**3c**	*i*-PrOH:H_2_O/1:2	8	83
4	**1d**	**3d**	H_2_O	5	89
5	**1e**	**3e**	DMFA: *i*-PrOH:H_2_O/1:1:1	5	61
6	**1f**	**3a**	i-PrOH:H_2_O/1:2	6	70

**Table 2:** Structure of compounds of the training and test sets.

No	Structure	No	Structure
**1**	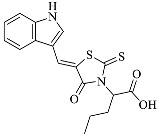	**8**	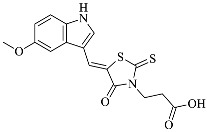
**2**	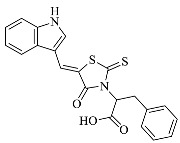	**9**	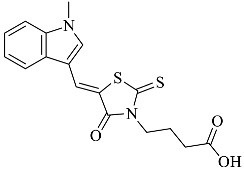
**3**	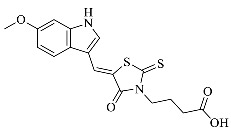	**10**	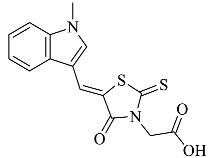
**4**	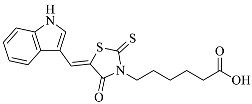	**11**	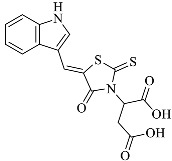
**5**	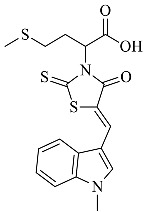	**12**	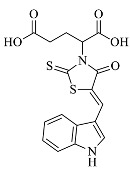
**6**	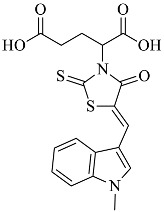	**13**	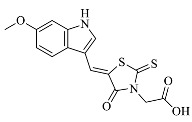
**7**	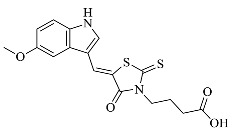	**14**	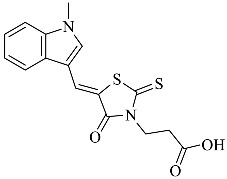

**Table 3:** Generated pharmacophores of the antibacterial activity against *S. aureus*.

Hypothesis	Features	Rank Score
Model-1	HHAAAADP	0.9345
Model-2	HHAAAADP	0.9338
Model-3	HHAAADP	0.9240
Model-4	HHAAADP	0.9197
Model-5	HHAAAAD	0.9191
Model-6	HHAAADP	0.9177
Model-7	HHAAADP	0.9125
Model-8	HHAAAAP	0.9035
Model-9	HHAAAAP	0.8975
Model-10	HHAAAP	0.8832

**Table 4:** Correlation between the biological activity and the fit value score against *S. aureus*.

Compounds	MIC (μM × 10^−^^2^)	−log MIC (μM)	Fit Value	Pred. −log MIC (μM)	Residual
**1**	0.56	2.251	123.59	1.994	−0.257
**2**	1.96	1.707	118.60	1.857	−0.150
**3**	1.99	1.701	117.83	1.836	−0.135
**4**	2.94	1.531	109.00	1.594	−0.063
**5**	3.69	1.432	109.00	1.594	−0.162
**6**	3.71	1.430	107.82	1.562	−0.132
**7**	3.98	1.400	108.25	1.573	−0.173
**8**	4.14	1.382	97.19	1.270	0.112
**9**	4.16	1.380	97.23	1.271	0.109
**10**	2.26	1.645	107.87	1.563	0.082
**11**	3.99	1.399	107.42	1.551	−0.212
**12**	7.68	1.114	86.95	0.990	0.124
**13**	8.61	1.064	85.78	0.958	0.106
**14**	8.66	1.062	85.10	0.939	0.123

**Table 5:** Experimental and estimated MIC values of tested compounds based on pharmacophore model-1 against *S. aureus*.

Compounds	Fit Value	Pred. −log MIC (μM)	MIC (μM × 10^−2^)	−log MIC (μM)	Residual
**5a**	106.27	1.508	3.78	1.422	0.158
**5b**	105.12	1.483	3.00	1.522	−0.039
**5c**	104.20	1.462	3.92	1.406	0.056
**5d**	87.66	1.097	7.03	1.153	−0.056
**5e**	78.83	0.901	8.51	1.070	−0.169
**5f**	86.25	1.065	7.64	1.116	−0.051
**5g**	97.89	1.345	3.94	1.404	−0.059
**5h**	97.12	1.306	3.55	1.449	−0.143
**5i**	77.70	0.876	7.89	1.102	−0.226
**5j**	88.23	1.109	5.36	1.270	−0.161
**5k**	88.62	1.118	5.57	1.254	−0.136
**5l**	87.49	1.093	5.16	1.287	−0.194

**Table 6:** Antibacterial activity of title compounds (MIC/MBC in µmol/mL × 10^−2^).

R.br		*B.c*	*M.f*	*S.a*	*L.m*	*En.cl*	*P.a*	*S.t*	*E. coli*
**5a**	**MIC**	7.57 ± 0.06	11.35 ± 0.1	3.78 ± 0.02	7.57 ± 0.1	3.78 ± 0.02	3.78 ± 0.02	11.35 ± 0.1	7.57 ± 0.02
	**MBC**	15.14 ± 0.1	15.14 ± 0.2	7.57 ± 0.06	15.14 ± 0.2	7.57 ± 0.06	7.57 ± 0.06	15.14 ± 0.2	15.14 ± 0.1
**5b**	**MIC**	8.19 ± 0.04	8.19 ± 0.04	3.00 ± 0.01	6.00 ± 0.04	3.00 ± 0.01	6.00 ± 0.02	6.00 ± 0.02	12.28 ± 0.1
	**MBC**	16.31 ± 0.1	16.31 ± 0.1	4.09 ± 0.02	8.19 ± 0.1	4.09 ± 0.02	8.19 ± 0.04	8.19 ± 0.04	16.31 ± 0.1
**5c**	**MIC**	7.84 ± 0.04	7.84 ± 0.04	3.92 ± 0.02	7.84 ± 0.05	3.92 ± 0.02	3.92 ± 0.02	5.75 ± 0.04	11.77 ± 0.2
	**MBC**	15.69 ± 0.08	15.69 ± 0.08	7.84 ± 0.04	15.69 ± 0.1	7.84 ± 0.04	7.84 ± 0.04	7.84 ± 0.08	15.69 ± 0.1
**5d**	**MIC**	7.03 ± 0.04	10.55 ± 0.1	7.03 ± 0.04	10.55 ± 0.1	7.03 ± 0.04	7.03 ± 0.04	7.03 ± 0.04	10.55 ± 0.2
	**MBC**	14.07 ± 0.2	14.07 ± 0.2	14.07 ± 0.2	14.07 ± 0.2	14.07 ± 0.1	14.07 ± 0.2	14.07 ± 0.2	14.07 ± 0.2
**5e**	**MIC**	8.51 ± 0.04	12.77 ± 0.1	8.51 ± 0.04	8.51 ± 0.08	3.12 ± 0.01	8.51 ± 0.04	8.51 ± 0.1	8.51 ± 0.08
	**MBC**	17.03 ± 0.08	17.03 ± 0.08	17.03 ± 0.08	17.03 ± 0.08	4.26 ± 0.02	17.03 ± 0.2	17.03 ± 0.08	17.03 ± 0.1
**5f**	**MIC**	7.64 ± 0.06	11.77 ± 0.1	7.64 ± 0.06	7.64 ± 0.06	5.75 ± 0.06	7.64 ± 0.06	11.77 ± 0.1	11.77 ± 0.1
	**MBC**	15.69 ± 0.2	15.69 ± 0.2	15.69 ± 0.1	15.69 ± 0.2	7.64 ± 0.08	15.69 ± 0.1	15.69 ± 0.1	15.69 ± 0.1
**5g**	**MIC**	3.94 ± 0.02	7.89 ± 0.06	3.94 ± 0.02	7.89 ± 0.02	3.94 ± 0.04	3.94 ± 0.02	7.89 ± 0.04	11.83 ± 0.1
	**MBC**	7.89 ± 0.04	15.77 ± 0.1	7.89 ± 0.08	15.77 ± 0.2	7.89 ± 0.08	7.89 ± 0.08	15.77 ± 0.2	15.77 ± 0.2
**5h**	**MIC**	7.31 ± 0.04	5.36 ± 0.04	3.55 ± 0.02	7.31 ± 0.04	3.55 ± 0.02	3.55 ± 0.02	7.31 ± 0.04	10.96 ± 0.1
	**MBC**	14.62 ± 0.2	7.31 ± 0.08	7.31 ± 0.08	14.62 ± 0.2	7.31 ± 0.04	7.31 ± 0.04	14.62 ± 0.2	14.62 ± 0.2
**5i**	**MIC**	7.89 ± 0.08	7.89 ± 0.08	7.89 ± 0.08	7.89 ± 0.04	7.89 ± 0.08	1.97 ± 0.04	7.89 ± 0.08	11.83 ± 0.1
	**MBC**	15.77 ± 0.2	15.77 ± 0.2	15.73 ± 0.2	15.77 ± 0.1	15.77 ± 0.2	3.94 ± 0.04	15.77 ± 0.1	15.7 ± 0.2
**5j**	**MIC**	7.31 ± 0.08	5.36 ± 0.04	5.36 ± 0.04	7.31 ± 0.04	7.31 ± 0.08	7.31 ± 0.08	7.31 ± 0.08	10.96 ± 0.1
	**MBC**	14.62 ± 0.2	7.31 ± 0.08	7.31 ± 0.08	14.62 ± 0.08	14.62 ± 0.1	14.62 ± 0.1	14.62 ± 02	14.62 ± 0.2
**5k**	**MIC**	7.57 ± 0.08	5.55 ± 0.04	5.57 ± 0.08	7.57 ± 0.08	7.57 ± 0.04	7.57 ± 0.04	7.57 ± 0.08	3.78 ± 0.04
	**MBC**	15.14 ± 0.2	7.57 ± 0.08	15.14 ± 0.2	15.14 ± 0.1	15.14 ± 0.1	15.14 ± 0.2	15.14 ± 0.2	7.57 ± 0.08
**5l**	**MIC**	7.03 ± 0.05	10.55 ± 0.1	5.16 ± 0.04	7.03 ± 0.08	5.16 ± 0.04	5.16 ± 0.04	7.03 ± 0.08	10.55 ± 0.1
	**MBC**	14.07 ± 0.1	14.07 ± 0.1	7.03 ± 0.08	14.07 ± 0.2	7.03 ± 0.08	7.03 ± 0.08	14.07 ± 0.02	14.07 ± 0.02
**Am**	**MIC **	24.80 ± 0.3	24.80 ± 0.2	24.80 ± 0.2	37.20 ± 0.4	24.8 ± 0.3	74.4 ± 0.9	24.80 ± 0.3	37.2 ± 0.4
	**MBC**	37.20 ± 0.4	37.20 ± 0.4	37.20 ± 0.2	74.40 ± 0.8	37.2 ± 0.3	124.0 ± 2	49.2 ± 0.6	49.2 ± 0.4
**Str**	** MIC **	4.30 ± 0.08	8.60 ± 0.1	17.20 ± 0.2	25.80 ± 0.4	4.3 ± 0.03	17.20 ± 0.3	17.2 ± 0.3	17.2 ± 0.3
	**MBC**	8.60 ± 0.1	17.20 ± 0.2	34.40 ± 0.4	51.60 ± 0.4	8.6 ± 0.06	34.40 ± 0.3	34.4 ± 0.3	34.40 ± 0.3

*B.c.*—*B. cereus*, *M.f.*—*M. flavus*, *S.a.*—*S. aurues*, *l.m.*—*L. monocytogenes*, *En.c.*—*En. cloacae*, *P.a.*—*P. aeruginosa*, *S.t.*—*S. typhimurium*, *E.c.*—*E. coli*.

**Table 7:** Antibacterial activity against resistant strains (µmol/mL × 10^−2^) and the effect on biofilm formation (inhibition percentage), * NE—no effect.

Compounds	Resistant Strains				Biofilm Formation	
		MRSA	*P.a.*	*E.c.*	MIC	0.5MIC
**5b**	**MIC**	136 ± 16	34 ± 2.7	136 ± 14.2	11.59	NE
	**MBC**	272 ± 33	68 ± 5.2	272 ± 31		
**5** **h**	**MIC**	122 ± 16.4	30.5 ± 2.8	122 ± 15.1	NE	NE
	**MBC**	244 ± 28	61 ± 7	244 ± 22.8		
**5g**	**MIC**	31.5 ± 2.7	31.5 ± 3.8	126 ± 14.7	18.19	NE
	**MBC**	63 ± 8.1	63 ± 4	252 ± 28		
**Streptomycin**	**MIC**	17.2 ± 2.1	8.6 ± 1.2	17.2 ± 2.1	71.94	55.42
	**MBC**	-	17.2 ± 1.4	34.4 ± 4.2		
**Ampicilline**	**MIC**	-	57.2 ± 6.4	57.2 ± 7.8	67.36	30.35
	**MBC**	/	/	/		

*E.c.*—*E. coli*, *P.a.*—*P. aeruginosa*.

**Table 8:** FICI indexes of combinations of selected compounds with streptomycin.

Compound	FICI
**5b**	1.5
**5g**	2
**5h**	1.5

**Table 9:** Antifungal activity of title compounds (MIC/MFC in µmol/mL × 10^−2^).

R.br		*A.f*	*A.v*	*A.o*	*A.n*	*T.v*	*P.o*	*P.f*	*P.v.c*
**5a**	**MIC**	3.78 ± 0.04	3.78 ± 0.04	3.78 ± 0.04	3.78 ± 0.04	2.02 ± 0.04	3.78 ± 0.04	3.78 ± 0.08	3.78 ± 0.04
	**MFC**	7.57 ± 0.08	7.57 ± 0.08	7.57 ± 0.08	7.57 ± 0.08	3.78 ± 0.04	7.57 ± 0.1	7.57 ± 0.1	7.57 ± 0.08
**5b**	**MIC**	8.19 ± 0.04	8.19 ± 0.08	3.00 ± 0.02	6.00 ± 0.08	3.00 ± 0.02	6.00 ± 0.08	6.00 ± 0.08	12.28 ± 0.1
	**MFC**	16.31 ± 0.2	16.31 ± 0.2	4.09 ± 0.03	8.19 ± 0.08	4.09 ± 0.03	8.19 ± 0.08	8.19 ± 0.08	16.31 ± 0.12
**5c**	**MIC**	15.69 ± 0.2	3.92 ± 0.06	2.09 ± 0.02	5.75 ± 0.04	2.88 ± 0.04	3.92 ± 0.08	3.92 ± 0.04	3.92 ± 0.08
	**MFC**	31.37 ± 0.4	7.84 ± 0.06	3.92 ± 0.03	7.84 ± 0.08	7.84 ± 0.08	7.84 ± 0.08	7.84 ± 0.1	7.84 ± 0.1
**5d**	**MIC**	29.31 ± 0.4	3.52 ± 0.04	1.88 ± 0.02	3.52 ± 0.04	1.88 ± 0.02	3.52 ± 0.02	3.52 ± 0.04	3.52 ± 0.04
	**MFC**	56.28 ± 1.1	7.03 ± 0.06	3.52 ± 0.02	7.03 ± 0.06	3.52 ± 0.04	7.03 ± 0.06	7.03 ± 0.06	7.03 ± 0.06
**5e**	**MIC**	34.05 ± 0.6	4.26 ± 0.08	3.12 ± 0.04	4.26 ± 0.05	3.12 ± 0.04	4.26 ± 0.08	6.24 ± 0.08	8.51 ± 0.08
	**MFC**	68.10 ± 0.8	8.51 ± 0.06	4.26 ± 0.05	8.51 ± 0.1	4.26 ± 0.05	8.51 ± 0.1	8.51 ± 0.08	17.03 ± 0.2
**5f**	**MIC**	15.69 ± 0.1	2.09 ± 0.04	2.09 ± 0.02	2.09 ± 0.04	2.09 ± 0.02	2.09 ± 0.02	2.09 ± 0.02	2.09 ± 0.04
	**MFC**	31.37 ± 0.4	3.92 ± 0.06	3.92 ± 0.04	3.92 ± 0.04	3.92 ± 0.06	3.92 ± 0.04	3.92 ± 0.04	3.92 ± 0.06
**5g**	**MIC**	3.94 ± 0.03	3.94 ± 0.04	2.10 ± 0.01	3.94 ± 0.06	2.10 ± 0.02	3.94 ± 0.06	3.94 ± 0.06	2.63 ± 0.02
	**MFC**	7.89 ± 0.08	7.89 ± 0.06	3.94 ± 0.06	7.89 ± 0.06	3.94 ± 0.06	7.89 ± 0.08	7.89 ± 0.06	7.89 ± 0.1
**5h**	**MIC**	14.62 ± 0.2	1.95 ± 0.01	1.95 ± 0.01	1.95 ± 0.02	0.97 ± 0.02	1.95 ± 0.02	1.95 ± 0.02	7.31 ± 0.1
	**MFC**	29.24 ± 0.4	3.55 ± 0.02	3.55 ± 0.04	3.55 ± 0.04	1.95 ± 0.04	3.55 ± 0.04	3.55 ± 0.06	14.62 ± 0.2
**5i**	**MIC**	3.94 ± 0.06	3.94 ± 0.06	2.10 ± 0.04	3.94 ± 0.08	2.10 ± 0.04	5.78 ± 0.08	3.94 ± 0.06	7.89 ± 0.06
	**MFC**	7.89 ± 0.08	7.89 ± 0.06	3.94 ± 0.06	7.89 ± 0.08	3.94 ± 0.06	7.89 ± 0.08	7.89 ± 0.06	15.77 ± 0.2
**5j**	**MIC**	14.62 ± 0.2	3.55 ± 0.06	3.55 ± 0.06	3.55 ± 0.04	5.36 ± 0.06	3.55 ± 0.05	3.55 ± 0.06	7.31 ± 0.06
	**MFC**	29.23 ± 0.4	7.31 ± 0.1	7.31 ± 0.1	7.31 ± 0.08	7.31 ± 0.1	7.31 ± 0.08	7.31 ± 0.08	14.62 ± 0.09
**5k**	**MIC**	3.78 ± 0.04	2.02 ± 0.04	2.02 ± 0.04	2.02 ± 0.04	2.02 ± 0.02	3.78 ± 0.02	3.78 ± 0.04	3.78 ± 0.04
	**MFC**	7.57 ± 0.08	3.78 ± 0.04	3.78 ± 0.04	3.78 ± 0.04	3.78 ± 0.08	7.57 ± 0.08	7.57 ± 0.1	7.57 ± 0.08
**5l**	**MIC**	2.58 ± 0.02	3.52 ± 0.02	2.58 ± 0.02	5.16 ± 0.06	3.52 ± 0.06	5.16 ± 0.06	3.52 ± 0.05	3.52 ± 0.04
	**MFC**	3.52 ± 0.04	7.03 ± 0.05	3.52 ± 0.05	7.03 ± 0.06	3.52 ± 0.04	7.03 ± 0.08	7.03 ± 0.08	7.03 ± 0.08
**Ketoconazole**	**MIC**	38.0 ± 1.2	285. ± 6.8	38.0 ± 1.2	38.0 ± 0.8	475.0 ± 5.8	380.0 ± 5.8	38.0 ± 1.2	38.00 ± 1.6
	**MFC**	95.0 ± 2.3	380. ± 8.4	95.0 ± 1.2	95.0 ± 0.6	570.0 ± 8.6	380.0 ± 4.8	95.0 ± 2.3	95 ± 2.6
**Bifonazole**	**MIC**	48.0 ± 2.2	48.0 ± 1.2	48.0 ± 2.8	48.0 ± 1.2	64.0 ± 2.8	48.0 ± 2.0	64.0 ± 1.2	48 ± 2.2
	**MFC**	64.0 ± 3.4	64.0 ± 0.8	80.0 ± 1.8	64.0 ± 2.3	80.0 ± 3.8	64.0 ± 1.6	80.0 ± 2.1	64 ± 3.4

*A.f.*—*A. fumigatus*, *A.v.*—*A. versicolor*, *A.o.*—*A. ochraceus*, *A.n.*—*A. niger*, *T.v.*—*T. viride*, *P.f.*—*P. funiculosum*, *P.o.*—*P. ochrochloron*, *P.v.c.*—*P. cyclpoium var verucosum*.

**Table 10:** Molecular docking binding affinities to antibacterial targets.

No	Est. Binding Energy (kcal/mol)					I-H *E. coli* MurB	Residues *E. coli* MurB
	*E. coli* Gyrase 1KZN	*S. aureus* Thymidylate Kinase 4QGG	*E. coli* Primase 1DDE	*E. coli* MurA JV4T	*E. coli* MurB 2Q85		
**5a**	−4.52	−1.91	−	−6.17	−8.59	2	Arg213, Ser228
**5b**	−6.25	−2.48	−1.26	−8.11	−12.57	3	Tyr157, Ser228, Lys261
**5c**	−4.27	−	−	−5.28	−9.45	2	Arg213, Ser228
**5d**	−2.74	−1.63	−2.44	−4.12	−7.02	1	Ser228
**5e**	−	−	−	−5.20	−6.97	1	Ser228
**5f**	−	−	−	−5.23	−7.12	1	Ser228
**5g**	−5.21	−1.69	−1.37	−6.76	−9.38	2	Ser228, Lys261
**5h**	−6.13	−1.47	−	−7.04	−10.28	2	Ser228, Asn232
**5i**	−	−1.32	−	−4.81	−7.55	2	Arg213, Ser228
**5j**	−5.33	−	−1.29	−6.05	−8.14	2	Arg213, Ser228
**5k**	−	−	−	−5.83	−7.75	2	Arg213, Ser228
**5l**	−	−2.55	−	−6.21	−8.82	2	Ser228, Asn232

**Table 11:** Molecular docking binding affinities to antifungal targets.

N/N	Est. Binding Energy (kcal/mol)		I-H	Residues CYP51 of *C. albicans*	Interactions with HEM601
	DNA TopoIV 1S16	CYP51 of *C. Albicans* 5V5Z			
**5a**	−1.55	−9.96	1	Tyr145	Negative Ionizable, Hydrophobic
**5b**	-	−8.54	2	Tyr135, Tyr145	Hydrophobic
**5c**	−3.34	−8.27	1	Tyr145	Hydrophobic
**5d**	−1.64	−7.83	−	−	Negative Ionizable, Hydrophobic
**5e**	-	−8.11	2	Tyr145, Thr315	Hydrophobic
**5f**	-	−9.93	2	Ser378, Met508	Hydrophobic
**5g**	−4.01	−10.80	1	Tyr145	Negative Ionizable, Hydrophobic
**5h**	−4.17	−9.81	1	Tyr145	Negative Ionizable, Hydrophobic
**5i**	−1.31	−9.71	1	Th315	Negative Ionizable, Hydrophobic
**5j**	−1.09	−8.54	1	Tyr145	Negative Ionizable, Hydrophobic
**5k**	−2.41	−13.01	3	Ser378, HEM601	Negative Ionizable, iron binding (Fe), hydrogen bond
**5l**	−4.25	−10.85	1	Tyr135	Negative Ionizable, Hydrophobic
**ketoconazole**	−	−8.23	1	Tyr64	Hydrophobic, aromatic
